# The RON2-AMA1 Interaction is a Critical Step in Moving Junction-Dependent Invasion by Apicomplexan Parasites

**DOI:** 10.1371/journal.ppat.1001276

**Published:** 2011-02-10

**Authors:** Mauld Lamarque, Sébastien Besteiro, Julien Papoin, Magali Roques, Brigitte Vulliez-Le Normand, Juliette Morlon-Guyot, Jean-François Dubremetz, Sylvain Fauquenoy, Stanislas Tomavo, Bart W. Faber, Clemens H. Kocken, Alan W. Thomas, Martin J. Boulanger, Graham A. Bentley, Maryse Lebrun

**Affiliations:** 1 UMR 5235 CNRS, Université de Montpellier 2, Montpellier, France; 2 Institut Pasteur, Unité d'Immunologie Structurale, CNRS, URA 2185, Département de Biologie Structurale et Chimie, Paris, France; 3 Center for Infection and Immunity of Lille, CNRS UMR 8204, INSERM U1019, Institut Pasteur de Lille, Université Lille Nord de France, Lille, France; 4 Department of Parasitology, Biomedical Primate Research Centre, Rijswijk, The Netherlands; 5 Department of Biochemistry & Microbiology, University of Victoria, Victoria, British Columbia, Canada; University of Geneva, Switzerland

## Abstract

Obligate intracellular Apicomplexa parasites share a unique invasion mechanism involving a tight interaction between the host cell and the parasite surfaces called the moving junction (MJ). The MJ, which is the anchoring structure for the invasion process, is formed by secretion of a macromolecular complex (RON2/4/5/8), derived from secretory organelles called rhoptries, into the host cell membrane. AMA1, a protein secreted from micronemes and associated with the parasite surface during invasion, has been shown *in vitro* to bind the MJ complex through a direct association with RON2. Here we show that RON2 is inserted as an integral membrane protein in the host cell and, using several interaction assays with native or recombinant proteins, we define the region that binds AMA1. Our studies were performed both in *Toxoplasma gondii* and *Plasmodium falciparum* and although AMA1 and RON2 proteins have diverged between Apicomplexa species, we show an intra-species conservation of their interaction. More importantly, invasion inhibition assays using recombinant proteins demonstrate that the RON2-AMA1 interaction is crucial for both *T. gondii* and *P. falciparum* entry into their host cells. This work provides the first evidence that AMA1 uses the rhoptry neck protein RON2 as a receptor to promote invasion by Apicomplexa parasites.

## Author Summary

Apicomplexa parasites are obligate intracellular pathogens causing severe diseases such as the deadly malaria or toxoplasmosis. Host cell invasion by these parasites involves the formation of a structure between the apex of the parasite and the host cell membrane called the moving junction (MJ), which is built upon collaboration between secretory organelles from the parasite that insert microneme protein AMA1 in the parasite plasma membrane and a complex of four rhoptry neck (RON2/4/5/8) proteins at the host cell plasma membrane. We have now identified a strong interaction between AMA1 and a C-terminal region of RON2, which is crucial for invasion. In spite of sequence variations in both proteins orthologs from distinct Apicomplexa, we could show that this interaction is functionally conserved and equally important for the invasive process by *Toxoplasma gondii* and *Plasmodium falciparum*. These findings open the way for therapeutic approaches that could interfere with key functions of parasitic MJ.

## Introduction

Apicomplexa parasites are responsible for important diseases affecting humans and animals, such as toxoplasmosis, malaria, neosporosis, coccidiosis and cryptosporidiosis. The most detrimental infection is malaria, caused by a parasite from the genus *Plasmodium*. It is transmitted by female *Anopheles* mosquito, placing about 40 per cent of the world's population at risk of high morbidity and mortality. Most Apicomplexa are obligate intracellular parasites. The cell invasion machinery of these parasites is highly conserved and involves a structure called the moving junction (MJ) formed between the parasite and host cell membranes [1]. The MJ moves from the apex to the posterior of the parasite, leading to its internalization into a new compartment called the parasitophorous vacuole (PV). The molecular components of the MJ have been recently deciphered [2], [3], showing that proteins unique to the Apicomplexa and generally conserved within the phylum are secreted from secretory organelles of the parasites called the rhoptries. Four rhoptry neck proteins from *Toxoplasma gondii* (RON2, RON4, RON5 and RON8) form a complex that is discharged during invasion [2], [3], [4] and targeted to the host cell membrane [5]. This complex has been found associated *in vitro* with the protein apical membrane antigen 1 (AMA1) [2], [6], [7], which is contained in another set of parasite secretory organelles called micronemes and discharged prior to the secretion of rhoptries during invasion [8]. AMA1 was first identified in *P. knowlesi* [9] and although its function was not clearly understood, it was shown to relocalize from the micronemes to the parasite surface and to be essential to parasite survival in *T. gondii* and *P. falciparum* [10], [11]. Numerous lines of evidence suggest that this conserved protein plays a central role in host cell invasion by Apicomplexa parasites; for instance, antibodies against AMA1 block parasite invasion [7], [12] and a conditional *T. gondii* AMA1 null mutant is unable to invade host cells [10]. During the past decade, AMA1 has become a major candidate for antimalarial vaccine development (see [13] for a review). In addition, the discovery of its association with the *T. gondii* MJ complex *in vitro* [2] and the demonstration of a similar complex in *Plasmodium* species [14], [15], [16] have reinforced interest in AMA1 as a target for therapeutic development. AMA1 vaccine candidates are currently undergoing clinical trials and their protective immune effector mechanism appears to be antibodies that block invasion [17]. Mimotope approaches are also being developed as therapeutic strategies. The recent resolution of x-ray crystal structures of *P. vivax* [18], *P. falciparum* [19] and *T. gondii* AMA1 [20] have revealed a conserved hydrophobic trough surrounded by polymorphic loops that are the target of *Plasmodium* growth-inhibitory antibodies [21], [22].

In *P. falciparum*, two recent publications [14], [23] showed that an antibody directed against AMA1 or a short peptide (R1 peptide) inhibited invasion by preventing PfAMA1 from interacting with the PfRON complex, without defining which RON is involved in this interaction. In *Toxoplasma*, we have shown that RON2 interacts directly with AMA1 *in vitro* and is exported with the other RONs to the host cell membrane [5], with RON4/5/8 being exposed to the cytoplasmic face of the host cell membrane and RON2 being predicted as a transmembrane (TM) protein. Although we could not determine the topology of RON2 once secreted and associated with the host cell membrane, we proposed a model whereby the parasite secretes and inserts the interacting components on both sides of the MJ, i.e. the plasma membranes of the host (RON2) and the parasite (AMA1) [5], respectively. In this model, RON4/5/8 anchor the junctional complex to the host cell cytoskeleton and/or restrict the access of host cell membrane proteins to the PVM.

Here we have further characterized the molecular interplay between AMA1 and RON2 in *T. gondii* and *P. falciparum* by identifying an interaction domain in RON2. We have also demonstrated the functionality and role of the RON2-AMA1 interaction during the invasion step. Our results open perspectives for new ways of interfering with parasite invasion, with a clear therapeutic potential against Apicomplexa.

## Results

### TgRON2 localizes at the MJ during invasion

**Figure 1:**
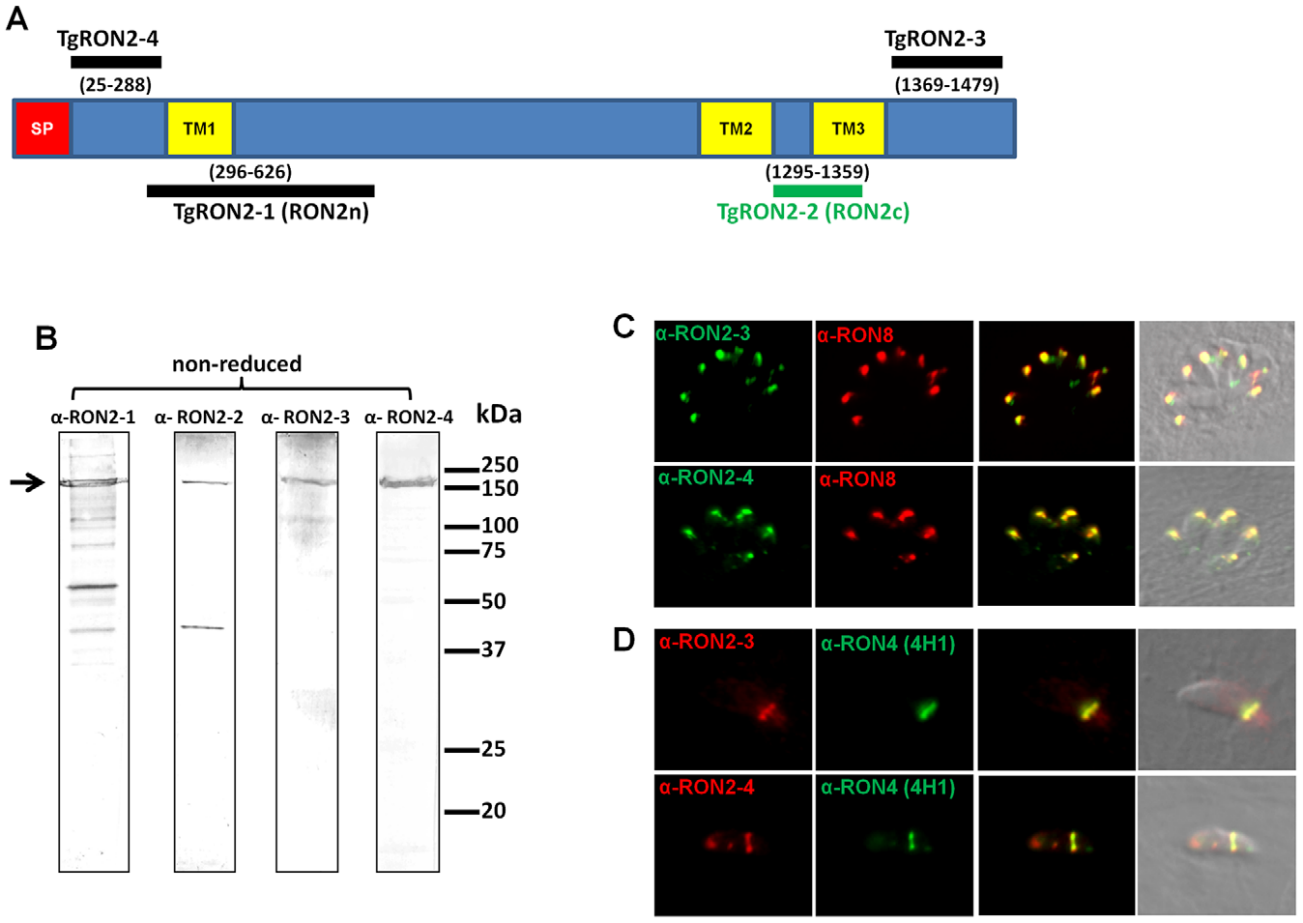
TgRON2 is present at the MJ during the entire invasion process. (A) Overall scheme of TgRON2 with its signal peptide (SP, red) and three predicted TM domains (TM, yellow). Bars indicate the region used to generate the antibodies (with amino acid positions numbered). (B) Western blot analysis of *T. gondii* tachyzoite lysates (∼5.10^6^ parasites per lane) with the different immune sera against TgRON2. The arrow marks the full length protein. (C) Co-localisation of TgRON2 with TgRON8 in the rhoptries of intracellular parasites, as revealed by the anti-RON2-3 and anti-RON2-4 antibodies. (D) Detection of TgRON2 at the MJ in invading parasites as revealed by the anti-RON2-3 and anti-RON2-4 antibodies.

While anti-RON4, anti-RON5 and anti-RON8 antibodies recognized the characteristic ring-shaped MJ during *T. gondii* invasion, we previously failed to detect TgRON2 (GenBank HQ110093) in the ring using two sera recognizing distinct regions of the protein [5]. TgRON2 was only observed at a very early stage, when the MJ appears as a dot at the apical contact with the host cell and when parasites were treated with cytochalasin D (Cyt-D) inducing a “static” junction that is not translocated to the posterior end of the tachyzoites [5]. We hypothesized that TgRON2 is engaged in intermolecular interactions precluding antibody binding. In order to re-examine the fate of TgRON2 during invasion, we generated new antisera directed against different domains of TgRON2. Our previous antisera were produced against recombinant proteins RON2n and RON2c (see Figure 1A), which we rename here as TgRON2-1 and TgRON2-2, respectively. TgRON2-1 is a His-tag recombinant protein that encompasses the first putative TM region (TM1) and TgRON2-2 is a GST-fusion protein corresponding to the peptide sequence located between the next two putative TM regions (TM2 and TM3), including 14 residues of TM3. We produced two additional His-tag proteins and specific sera. TgRON2-3 corresponds to the extreme C-terminal part of the protein after TM3 and TgRON2-4 extends from the first residue after the signal peptide to the residue preceding TM1. The specificity of these sera was assessed by Western blot, and all revealed a main protein at around 150 kDa (Figure 1B), which is the apparent size of RON2 in non reduced conditions [5]. Anti-RON2-1 and anti-RON2-2 antibodies [5] are revealing additional bands that could be unspecific degradation products or cross-reacting proteins, as new antibodies generated against N- and C- terminal parts confirm that there is no processing product visible in non-reduced conditions (Figure 1B). Immunofluorescence assay (IFA), revealed a perfect co-localisation with TgRON8 in the rhoptries of intracellular parasites (Figure 1C). On invading parasites, we found that both anti-RON2-3 and anti-RON2-4 antibodies recognized exclusively the characteristic ring-shaped MJ (Figure 1D) and the residual posterior dot that persists after invasion (Figure S1). This definitively demonstrates that TgRON2 is present at the MJ during the entire invasion process, as we previously hypothesized.

### Anti-RON2-2 serum fails to co-immunoprecipitate TgAMA1

**Figure 2:**
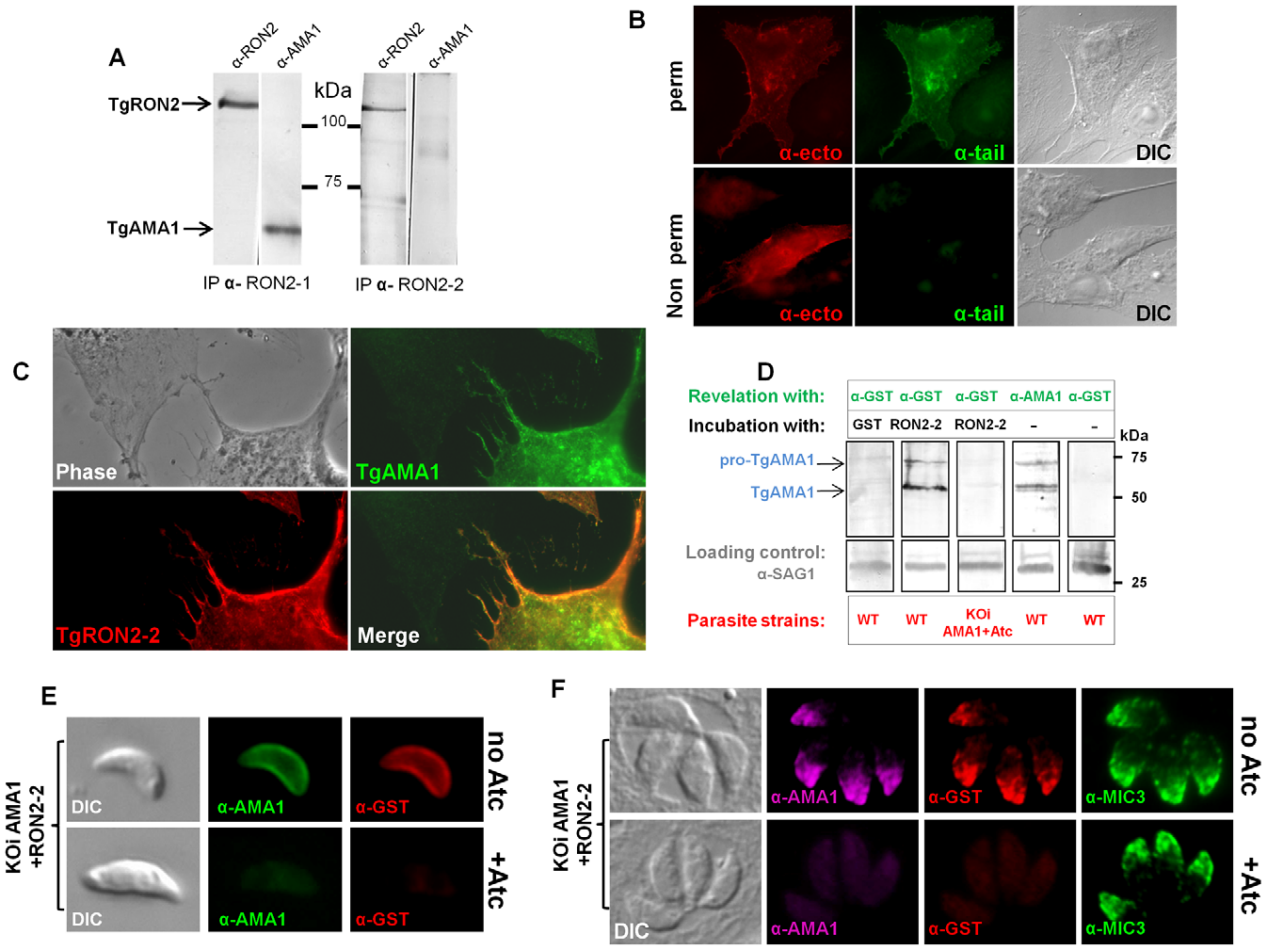
Mapping the interactions between TgRON2 and TgAMA1. (A) Co-immunoprecipitation of TgAMA1 and TgRON2 after 0.6% SDS lysis and Triton X100 renaturation of tachyzoite extract: anti-RON2-1 allows the recovery of both proteins, while anti-RON2-2 only recovers TgRON2. (B) Transiently transfected BHK-21 cells express TgAMA1 at their surface in its native conformation, with the ectodomain exposed outside (revealed with antibody B3.90, “ -ecto”) and the cytosolic tail only detected after permeabilization (revealed with antibody CL22, “ -tail”). (C) TgAMA1-expressing BHK-21 cells were incubated with 0.1 µg/ml TgRON2-2 (a GST-fusion protein), washed and the binding of recombinant TgRON2 fragment to TgAMA1-expressing cells was revealed with anti-GST antibody. Untransfected BHK-21 cell on the left showed no TgRON2 binding. (D) Far Western blot analysis of TgRON2-2 binding to native TgAMA1. Extracts of tachyzoites expressing wild-type levels of AMA1 (WT) or depleted for this protein (KOiAMA1+Atc) were separated by SDS-PAGE and transferred on a nitrocellulose membrane, which was incubated with GST alone or TgRON2-2 and revealed with anti-GST. Incubation with TgRON2-2 revealed a band corresponding to the size of TgAMA1, as shown by specific antibodies and absent from the AMA1-depleted cell line extracts, while anti-GST antibody did not react with tachyzoites. Anti-SAG1 was used as a loading control. (E) TgRON2-2 binds specifically to surface-exposed TgAMA1. Fixed extracellular conditional KOi AMA1 tachyzoites induced (+Atc) to repress AMA1 expression, or non-induced (no Atc), were incubated with TgRON2-2, which was then revealed with anti-GST antibody. TgAMA1 was revealed with anti-ectodomain B3.90 antibody. (F) TgRON2-2 binds specifically to TgAMA1 in micronemes. Fixed intracellular induced or non-induced conditional KOi AMA1 tachyzoites were permeabilized and incubated with TgRON2-2, which labelled micronemes only when TgAMA1 was expressed. MIC3, an unrelated microneme marker, was used as a control.

We have previously shown that while NP40 lysis does not disrupt the complex formed by the TgRONs and TgAMA1 (GenBank AF010264) [5], SDS lysis destabilizes it, although allowing some TgAMA1-TgRON2 re-association after Triton-X100 renaturation. This suggests a strong specific interaction between these two partners (Figure 2A). We have now observed that the anti-RON2-2 antibodies fail to immunoprecipitate the complex (RON2 included) after NP40 lysis (data not shown). By contrast anti-RON2-2 antibodies immunopurified TgRON2 after SDS lysis, but this time failed to co-immunopurify TgAMA1 (Figure 2A), suggesting that only free TgRON2 was bound and that the region corresponding to TgRON2-2 was not accessible to these antibodies when linked to TgAMA1. Overall, these data suggest that the region of TgRON2 that binds TgAMA1 is located between amino acid 1295 and 1359 (Figure 1A). Interestingly, TgRON2-2 is part of the C-terminal region of RON2, which is the most conserved between apicomplexan RON2 orthologs (Figure S2), suggesting a conserved function for this region.

### The TgRON2-2 recombinant protein binds specifically to TgAMA1

We developed a heterologous expression system that displays TgAMA1 on the surface of BHK-21 mammalian cells with a type 1 TM topology, to investigate whether TgRON2-2 produced as a GST-fused recombinant protein could interact specifically with TgAMA1 in the absence of the other RON members of the MJ complex. This topology mimics the one adopted by TgAMA1 once secreted onto the parasite surface [6], and allowed us to perform binding assays by incubating these AMA1-expressing cells with TgRON2-2.

We thus constructed a plasmid encoding the TgMIC3 signal sequence, previously shown to drive ectopic proteins in the mammalian secretory pathway [24], directly fused to the mature TgAMA1 coding sequence. Two potential N-glycosylation sites (Asn-X-Ser/Thr) were mutated, since the native protein is not glycosylated. The resulting pcDNA3-TgAMA1 plasmid was transiently transfected in BHK-21 cells and expression of TgAMA1 was analyzed by IFA. Using antibodies recognizing either the cytoplasmic C-terminal tail of TgAMA1 (α-tail) or a monoclonal antibody against the ectodomain (Mab B3.90, α-ecto) on permeabilized cells, they labeled the whole cell together with a perinuclear and vesicular pattern, suggesting that TgAMA1 was present in the secretory pathway and exposed at the cell surface (Figure 2B). Without permeabilization, only the α-ecto antibody reacted, revealing TgAMA1 at the surface of transfected cells (Figure 2B). The C-terminal part of AMA1 was thus not accessible in these conditions, showing that TgAMA1 had adopted a type 1 TM insertion in BHK-21 plasma membrane, mimicking its native topology at the parasite surface.

We then investigated whether recombinant TgRON2-2 would bind BHK21 cells expressing TgAMA1 by incubating them with the protein. Since TgRON2-2 is a GST-fusion protein, incubation with GST alone was used as control. After several washes, the cells were fixed, permeabilized and processed for IFA. Transfected cells were detected using the TgAMA1 α-tail antibody, and binding of TgRON2-2 or GST was analyzed with anti-GST. As a control, BHK21 cells were transfected with a plasmid promoting surface expression of TgMIC8 [24], another microneme protein expressed at the parasite surface during invasion but absent from the MJ complex [25]. While GST alone was never detected (Figure S3A), TgRON2-2 was systematically and specifically detected at the surface of TgAMA1-transfected cells (Figure 2C), but did not bind to unrelated TgMIC8-expressing cells (Figure S3B), confirming a specific interaction between TgRON2-2 and TgAMA1.

In order to verify if TgRON2-2 also bound native TgAMA1, we tested the interaction by two other different approaches. In the first (far western), tachyzoite proteins were separated by SDS-PAGE, transferred to a nitrocellulose membrane, which was then incubated with TgRON2-2 or GST and revealed using anti-GST serum. As expected only TgRON2-2 bound to a protein band, which co-migrated with TgAMA1 (Figure 2D). Moreover, we used a conditional TgAMA1 knock-out *T. gondii* strain expressing an anhydrotetracycline (Atc)-regulated exogenous copy (KOi AMA1) [10], grown for 24h with Atc to turn down the expression of TgAMA1. When lysates from these parasites were used, TgRON2-2 was not found to bind anymore (Figure 2D), confirming a specific interaction between TgRON2-2 and TgAMA1. Also, we analyzed TgRON2-2 binding to TgAMA1 *in situ* by incubating TgRON2-2 with extracellular parasites expressing, or not, TgAMA1 at their surface, as, once secreted from the micronemes, TgAMA1 relocalizes to the parasite surface. TgRON2-2 was found to bind to the surface of TgAMA1-expressing parasites, while it was undetectable in Atc-induced parasites depleted from TgAMA1 (Figure 2E). GST, used as a control, showed no binding (data not shown). Furthermore, we assessed whether TgRON2-2 was able to specifically bind to the micronemal pool of TgAMA1 present within the parasites. HFF were infected with KOi AMA1 parasites [10] and grown without or with Atc to express or turn down the expression of TgAMA1, respectively. Both infected cell monolayers were fixed, permeabilized and incubated with TgRON2-2 before revelation with anti-GST. Remarkably, TgRON2-2 labeled the micronemes, co-localizing perfectly with anti-AMA1 staining and this signal was absent from TgAMA1-depleted parasites, while microneme marker MIC3 was detectable in both conditions (Figure 2F).

### The N-terminal domain of TgRON2 is exposed at the cytosolic face of host cell membrane

We have previously shown that the MJ RON complex is targeted to the host cell membrane during invasion, with RON4, RON5 and RON8 being exposed on the cytosolic face of the membrane, but we failed to determine the topology of RON2 using anti-RON2-1 and RON2-2 antibodies [5]. Indeed, several TM predictions using bio-informatics tools (Table S1) modelled TgRON2 as a TM protein bearing three TM domains (Figure 1A). However, the exact number of these domains and the topology of the protein at the plasma membrane could not be resolved. We aligned the RON2 orthologs from different Apicomplexa species to further refine a model of RON2 topology (Figure S2) using several TM prediction programs to assess the conservation of the TMs. The N-terminal region of RON2 is clearly quite variable. TM1 is not conserved across all orthologs (Table S1) and displays the lowest confidence index in predictions, suggesting that this polypeptide sequence may not constitute a real TM domain. Another hydrophobic amino acid-rich N-terminal region is quite conserved among Apicomplexa RON2 orthologs (residues 404 to 424 in TgRON2) but does not yield a consistent TM prediction (data not shown). By contrast, TM2 and TM3 are consistently predicted in different Apicomplexa RON2 orthologs.

**Figure 3:**
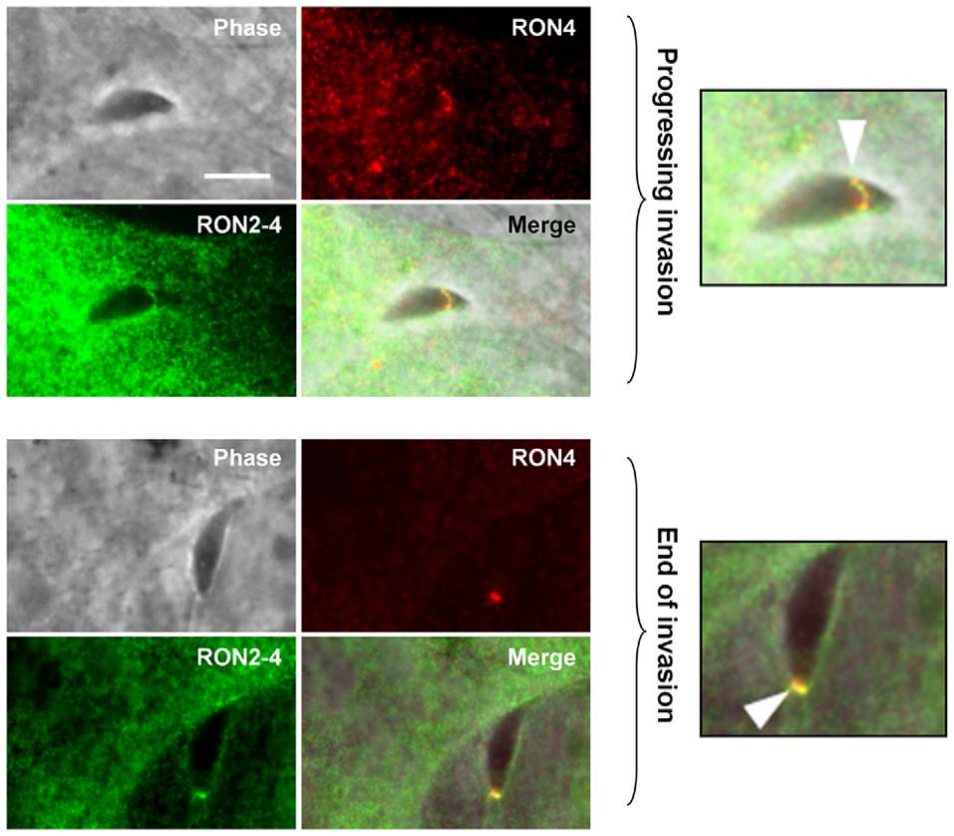
TgRON2 exposes at least one segment on the cytosolic side of the host cell plasma membrane. HFF cells were pre-loaded with antibodies directed against TgRON2-4 and were pulse-infected for 2.5 min, followed by IFA. The upper images show a progressing invasion while the lower images show a terminating invasion, in both cases the arrowhead indicates the MJ and magnifications are shown on the right. Scale bar = 5µm.

The fact that TgRON2-2 was able to bind TgAMA1 strongly suggested that the segment between TM2 and TM3 was expressed on the host cell surface. We thus tried to examine the topology of TgRON2 by expression of the full length TgRON2 protein with its own signal peptide in mammalian cells, using the pcDNA3.1 vector (Invitrogen). Attempts were also made with different truncated RON2 versions, including each of the putative transmembrane domains (TM1, amino acids 318–340; TM2, amino acids 1277–1296; TM3, amino acids 1346–1368) preceded by TgMIC3 signal peptide sequence [24]. In all cases, the expressed proteins were detected in the endoplasmic reticulum of transfected cells but were not trafficked to the plasma membrane, which prevented us from using this heterologous system to study TgRON2 topology. We therefore re-examined the topology of TgRON2 by an approach we used previously [5] to localize RON4, RON5 and RON8, using the newly generated anti-RON2 antibodies that allow detection of the protein at the MJ (Figure 1C). To this end, we pre-loaded the host cells with anti-RON2-3 or anti-RON2-4 antibodies using mechanical glass beads loading and subsequently infected the cells with *Toxoplasma* tachyzoites. The cells were then fixed during invasion, permeabilized and subjected to fluorescent secondary antibody detection. As we have shown before, RON4 is detected at the MJ in these conditions, as it is exposed on the cytosolic face of the host cell and is thus accessible to the antibodies, while RON2 could not be detected with the anti-RON2-2 antibodies (Figure S4). However, by pre-loading host cells with anti-RON2-4 we clearly detected TgRON2 at the progressing MJ, or after completion of invasion (Figure 3), while no signal was detected without permeabilization (data not shown). Although some parasites displayed signals resembling the MJ, no clear and consistent labelling was detected by preloading with anti-RON2-3 (Figure S4), preventing us from determining whether this part of the protein is exposed or not on the cytosolic side of the membrane. This result demonstrated that at least the N-terminal region of TgRON2 is exposed on the cytosolic side of the host cell membrane, while TgRON2-2 most likely interacts with AMA1 on the outside of host cell. In conclusion, even though we have not clearly identified the TM domain(s) of RON2, our results show that TgRON2 expressed segments on both sides of the host cell membrane and that it is therefore an integral membrane protein.

### The TgRON2-2 recombinant protein inhibits *Toxoplasma* invasion through interaction with AMA1

**Figure 4:**
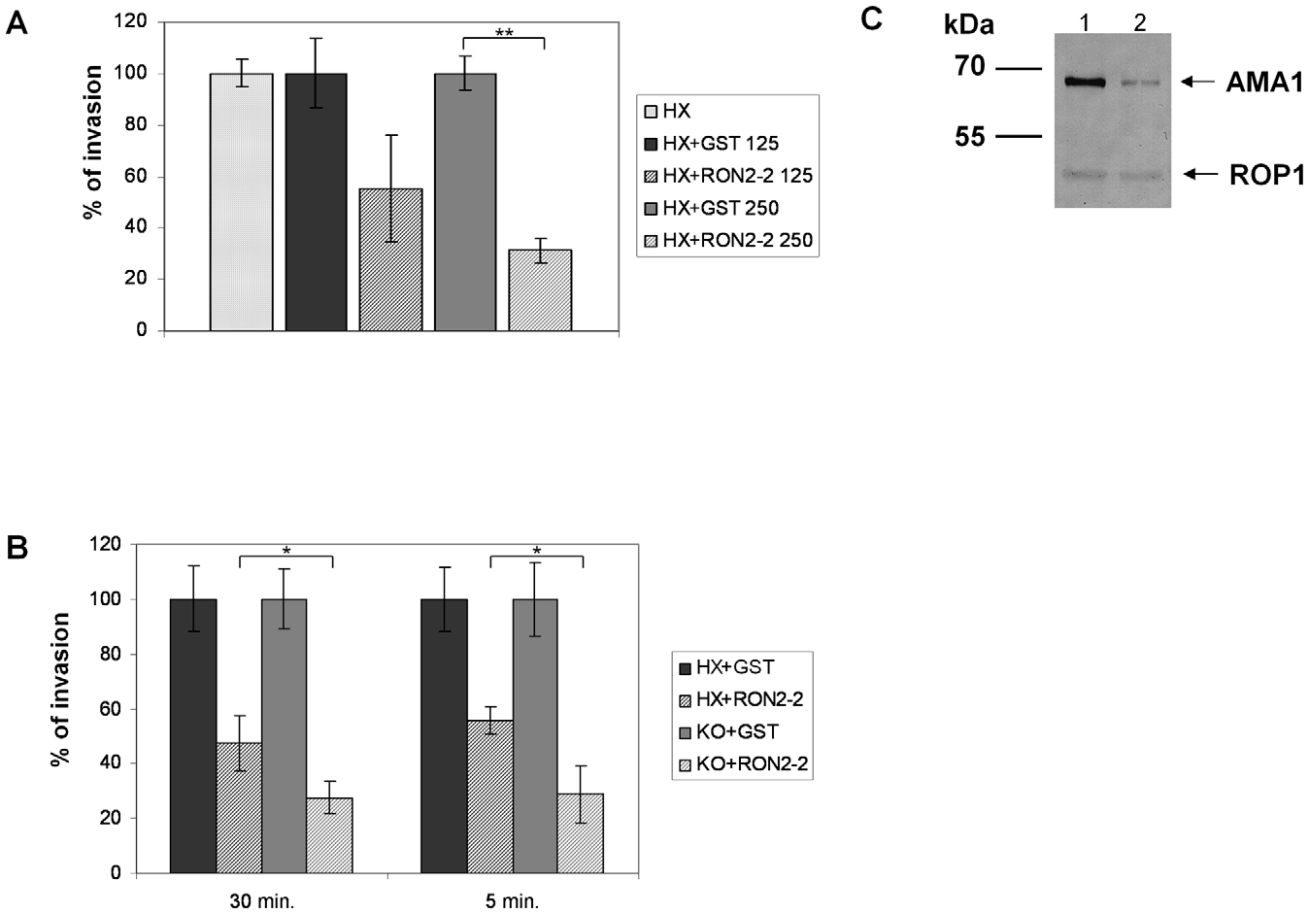
TgRON2-2 inhibits *T. gondii* invasion by interfering with endogenous RON2-AMA1 interaction. (A) Extracellular tachyzoites were pre-incubated with either GST or TgRON2-2 at 125 µg/ml (3.75 µM) or 250 µg/ml (7.5 µM), before being added to confluent HFF monolayers for 30 min. Significant decrease in *T. gondii* invasion (*t*-test, ** p<0.01) was observed in the presence of TgRON2-2 at a concentration of 250 µg/ml. (B) Decreasing TgAMA1 expression at the surface is correlated with an enhanced effect of TgRON2-2 on invasion. RH *hxgprt-* and KOi AMA1 strains were compared for their invasion efficiency in the presence of GST or TgRON2-2 at 200 µg/ml (6 µM). The invasion process was stopped by cell fixation after 30 min or 5 min. In both cases, KOi AMA1 shows a more pronounced invasion defect in presence of TgRON2-2 compared to RH *hxgprt-* (*t*-test, * p<0.05). Values represent means ± standard deviations (SD), n = 3, from a representative experiment out of 4 independent assays. (C) Western blot analysis of TgAMA1 expression levels in RH strain (1) or in the KOi AMA1 strain grown in absence of Atc (2). Anti-ROP1 antibody was used as a loading control.

Our previous study suggested a model of MJ organization where the parasite exports sequentially a ligand (AMA1) at the parasite cell surface and then its own receptor (RON2) at the host cell membrane. Since recombinant TgRON2-2 interacts directly with TgAMA1, we hypothesised that it competitively inhibits the interaction between endogenous TgAMA1 and TgRON2 and thus the invasion process. We therefore quantified the invasion efficiency of *T. gondii* tachyzoites in HFF cells in the presence of TgRON2-2 or GST. Extracellular parasites were pre-incubated with recombinant proteins for 30 min before performing the invasion of the host cells, still in the presence of recombinant proteins. Recombinant TgRON2-2 inhibited the invasion by 69% at 250 µg/ml compared to GST control (Figure 4A). If TgRON2-2 indeed acts as a competitive inhibitor of the RON2-AMA1 interaction on the parasite/host cell interface, we reasoned that the inhibitory effect would increase in a strain expressing less AMA1. Accordingly, we took advantage of the *T. gondii* KOi AMA1 strain described above that displays no invasion defect in absence of Atc despite expressing only ∼10% of the wild type TgAMA1 level (Figure 4C) [10]. We therefore compared the inhibitory effect of TgRON2-2 on KOi AMA1 with the wild type. As shown in Figure 4B, incubation of KOi AMA1 parasites with TgRON2-2 during the 30 minutes invasion time induced a significantly more pronounced decrease in invasion efficacy compared to wild-type. Similar results were obtained when the invasion time was reduced to 5 minutes (Figure 4B).

The invasion process is a multistep mechanism starting with an attachment step; we thus tested the ability of TgRON2-2 to inhibit the attachment of tachyzoites on HFF cells fixed with glutaraldehyde, which blocks parasite penetration by rigidifying the target cells. As shown in Figure S5, we did not observe any effect of TgRON2-2 on attachment of KOi AMA1 or wild type strains. Taken together, these results show that the inhibitory effect of TgRON2-2 takes place through perturbation of the TgAMA1-TgRON2 interaction, which is a critical step for the invasion process occurring after the initial attachment step.

### The RON2-AMA1 interaction and its role during invasion are evolutionarily conserved

**Figure 5:**
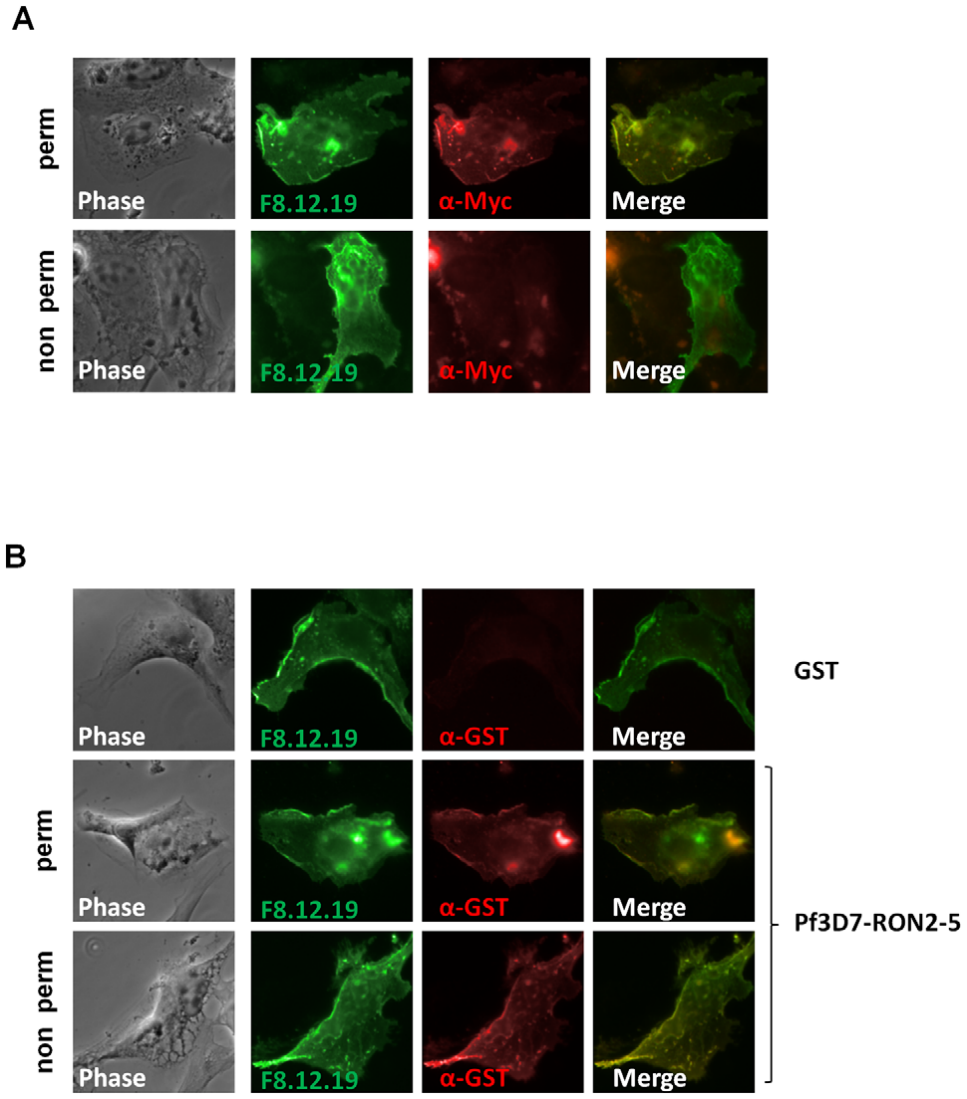
RON2-AMA1 interaction is conserved in *P. falciparum*. (A) Pf3D7-AMA1 displays a type I TM topology at the plasma membrane of BHK-21 cells. PfAMA1 transfected BHK-21 cells were subjected to IFA. Mouse MAb F8.12.19 recognizes extracellular PfAMA1 domain III that is accessible with or without permeabilization, whereas anti-Myc antibody labels the intracellular C-terminal Myc tag in permeabilized cells only. (B) PfAMA1 transfected BHK-21 cells were incubated with 20 µg/ml of GST or PfRON2-5. Recombinant proteins were detected using anti-GST antibody.

Major components of the MJ are conserved between most Apicomplexa species and are recovered as a complex by immunopurification, suggesting a conserved function for the complex. Conservation of the RON2-AMA1 interaction was tested by expression of the region located between predicted TM2 and TM3 of *P. falciparum* 3D7 RON2 (GenBank AAN37108), named PfRON2-5 (see Figure S2) and assay of its binding capacity to *P. falciparum* 3D7 AMA1 (GenBank AAN35928) using the mammalian expression system described above. Correct TM 1 topology of PfAMA1 at BHK-21 cells plasma membrane was first verified by IFA performed with or without permeabilization (Figure 5A). Indeed, the AMA1-fused C-terminal myc epitope was only detected in permeabilized cells, while MAb F8.12.19, which recognizes extracellular PfAMA1 domain III [26], gave a similar staining with or without detergent treatment. Incubation of PfAMA1-transfected cells with PfRON2-5 allowed detection of PfRON2-5 binding to AMA1, but not of the GST control (Figure 5B), thus demonstrating that RON2-AMA1 interaction is conserved in Apicomplexa parasites.

**Figure 6:**
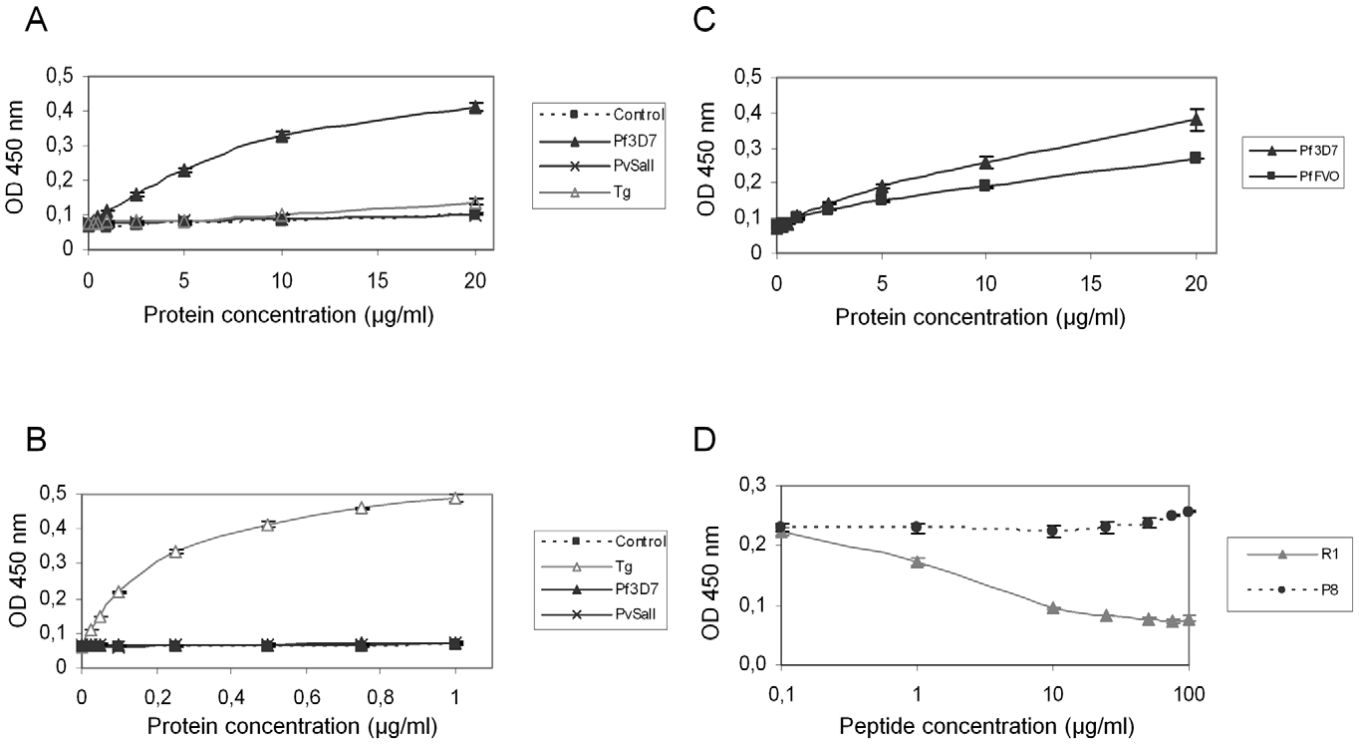
Intra-species specificity of RON2-AMA1 interaction. ELISAs were carried out with increasing concentrations of Pf3D7-RON2-5 (A) or TgRON2-2 (B) and recombinant AMA1 proteins from Pf3D7, PvSalI, TgRH, or PfFVO (C) strains coated at 1 µg/ml. GST alone was used as a control. (D) Competitive ELISAs were performed using 20 µg/ml Pf3D7-RON2-5, 1 µg/ml Pf3D7-AMA1 and increasing concentrations of R1 peptide, or P8 control peptide. Values represent means ± SD, n = 2, from a representative experiment out of 4 independent assays.

AMA1 orthologs display the same folding pattern between species but extensive polymorphism has been described [27]. Figure S2 shows that sequence polymorphism also occurs in the region located between TM2 and TM3 of RON2 between Apicomplexa genera. The possibility of conservation of the RON2-AMA1 interaction between species was thereafter tested by Enzyme-Linked Immuno-Sorbent Assay (ELISA). To this end, we used recombinant AMA1 from *T. gondii* [20], *P. falciparum* [28] and *P. vivax* (GenBank CAA76546) [29] coated on ELISA plates and incubated them with increasing concentrations of TgRON2-2 or PfRON2-5. We observed a significant binding of PfRON2 to PfAMA1 (Figure 6A) and TgRON2 to TgAMA1 (Figure 6B), but no interspecies or intergeneric cross-binding was seen. However, PfRON2-5 was able to bind both *P. falciparum* 3D7-AMA1 and *P. falciparum* FVO-AMA1 (GenBank CAC05390) (Figure 6C), indicating that the RON2 and AMA1 proteins have diverged between species but that the RON2-AMA1 interaction is maintained intraspecies, highlighting the conserved, and probably crucial, role of this interaction for the invasion process.

A recent study showed that the AMA1-binding R1 peptide [30] blocked invasion by preventing AMA1 interaction with the PfRON complex [23] and that its binding region spanned the full length of the AMA1 hydrophobic trough. Here, we demonstrated that incubation with R1 prevents the binding of PfRON2-5 to PfAMA1 in a dose-dependent fashion, while control peptide P8 did not prevent this association (Figure 6D). As expected, the competitive effect of R1 was restricted to *P. falciparum*, as it was not able to inhibit the TgAMA1-TgRON2 interaction (data not shown). The competitive inhibitory effect of R1 confirms the importance of the hydrophobic trough of AMA1 in the functional interaction with RON2 and as a target for inhibitory molecules.

**Figure 7:**
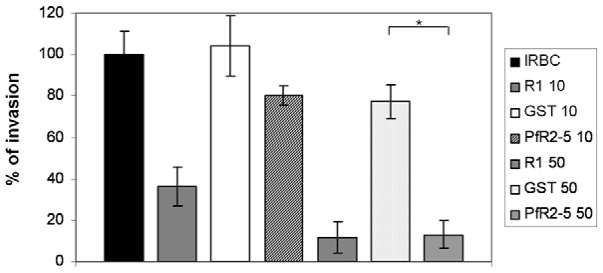
PfRON2-5 inhibits invasion of red blood cells by *Plasmodium* merozoites. Inhibition of invasion assays were performed with 2% of mature schizonts parasites that were incubated with 10 µg/ml (4.3 µM) or 50 µg/ml (21 µM) of R1 peptide or molar equivalent concentrations of PfRON2-5 or GST proteins. Parasitemia of control infected red blood cells (IRBC) 16 hours post-invasion was used as the 100% of invasion reference. R1 peptide inhibits parasites invasion, and so does PfRON2-5 at the highest concentration compared to GST control (*t*-test, * p<0.05). Values represent means ± SD, n = 4, from a representative experiment out of 3 independent assays.

AMA1-binding R1 peptide inhibits host cell invasion by *Plasmodium* merozoites and we have demonstrated that TgRON2-2 displays a similar inhibitory effect on *T. gondii* tachyzoites (Figure 4). Therefore, we next tested if PfRON2-5 could also prevent *Plasmodium* merozoites entry into red blood cells. Highly synchronized *P. falciparum* 3D7 mature schizonts were incubated either with R1 peptide as a positive control of invasion inhibition, or PfRON2-5, or GST as a negative control. Equal molar concentrations of recombinant proteins were used relative to R1 peptide concentration. Blood smears were performed 16 hours post-invasion and counting of ring-stage parasites allowed us to evaluate the invasion efficiency. As shown in Figure 7, R1 peptide was able to decrease *P. falciparum* 3D7 invasion capacities to 37% at 10 µg/ml (4.3 µM) and to 11% at 50 µg/ml (21.7 µM), which is consistent with what has already been reported [30]. Although addition of PfRON2-5 at low concentration did not significantly affect merozoites re-invasion compared to GST control, use of a higher concentration led to a drastic inhibition of invasion, in the same range as with R1 peptide. In addition, we confirmed that the AMA1-RON2 interacting domains have evolved divergently, as PfRON2-5 was not able to inhibit the invasion process of *T. gondii* parasites, contrasting with the strong inhibitory effect observed with TgRON2-2 or TgRON2-5 (Figure S6). TgRON2-5 is a shorter construct corresponding exactly to the domain located between TM2 and TM3, similar to the one produced for *Plasmodium* (Figure S2), and that displays affinity to TgAMA1 (Figure S6). Overall, this shows that, not only PfRON2-5 can bind to PfAMA1, but it can also interfere with PfAMA1-PfRON2 interaction and block *Plasmodium* invasion similarly to what we have observed for *Toxoplasma*. Moreover, we have defined a similar minimal region located between putative TM2 and TM3 both in *P. falciparum* and *T. gondii* RON2, which is only able to interact specifically with AMA1 from the same species.

## Discussion

Host cell invasion, which is a critical process in *T. gondii*'s life cycle, is highly dependent on the successive exocytosis of two secretory organelles named micronemes and rhoptries, and the formation of the MJ that anchors the parasite to the host cell surface. The discovery of a rhoptry neck proteins complex (RON2/4/5/8) localized at the MJ during invasion, and association of this complex with the microneme protein AMA1, strongly suggests the existence of a concerted action between secreted parasitic proteins originating from microneme and rhoptry organelles in the invasion step, and probably in the MJ formation. However, the exact function and the interactions between each protein composing this molecular complex are largely unknown at present. Here, we have demonstrated that there is a tight and specific interaction between rhoptry neck protein RON2 and micronemal protein AMA1 in both *Toxoplasma* and *Plasmodium*, and have shown that the interaction of these two proteins at the parasite/host cell interface is essential for the invasion process.

We have previously demonstrated a strong *in vitro* interaction between native RON2 and AMA1 from *Toxoplasma* using immunoprecipitation experiments [5]. In the present study, we found that an antibody directed against a specific RON2 sequence located between two putative C-terminal TM domains specifically disrupts this interaction, suggesting that it competes with a zone of interaction between the two proteins. When produced as a GST-tagged recombinant protein, this domain showed significant binding to recombinant AMA1 by ELISA. Moreover, TgRON2-2 was able, in formaldehyde-fixed parasites, to bind specifically to micronemes where AMA1 is located, or to the surface of mammalian cells expressing AMA1 at their plasma membrane, giving additional evidence of the interaction of this region of RON2 with AMA1. More importantly, the use of recombinant TgRON2-2 protein in invasion inhibition assays allowed us to show the functional importance of the RON2-AMA1 interaction during this process. Indeed, incubation of parasites with TgRON2-2 inhibited invasion probably by competition with native RON2 for AMA1 interaction. Consistent with this conclusion, the inhibitory effect was more pronounced when a mutant that weakly expresses AMA1 was used. These results show for the first time that the RON2-AMA1 interaction is of significant importance for the invasion process and that this interaction takes place at the host-parasite interface once secreted from their respective organelles, yet this AMA1 function does not seem to be involved in the early attachment steps, as suggested before [10].

While previously we were only able to detect RON2 at the nascent MJ [5], in the present study we have clearly demonstrated the presence of RON2 at the progressing MJ in co-localisation with other RON partners. RON2 is therefore present at the host cell–parasite interface during the complete invasion process. In the model we proposed for the MJ organisation, AMA1 is a TM protein located on the parasite side, while RON2 (which has several predicted TM domains), is its binding partner on the host cell side after secretion by tachyzoites [5]. Using a comparative analysis of TM segments in multiple apicomplexan RON2 orthologs with several prediction algorithms, we found that the first predicted TM is not well conserved while the two TM encompassing the AMA1-interacting fragment are generally present. The use of a specific antiserum (anti-RON2-4) with permeabilized host cells allowed us to unambiguously determine that the N-terminal portion of RON2 was exposed inside, although we were unable to definitively assign the location of the C-terminus of the protein using the anti-RON2-3 antibody. This antiserum, although relatively efficient at detecting TgRON2 by Western blot analysis (Figure 1B) and at the MJ by IFA after fixation and permeabilization (Figure 1C), might be reacting poorly in this assay if the C-terminal region of TgRON2 is engaged in an interaction with an intracellular protein partner during MJ formation that would preclude recognition by the antibody in the particular conditions used for this assay (i.e. antibody reacting directly on forming MJ structure in live parasites entering host cell, fixation and permeabilization being performed afterwards).

**Figure 8:**
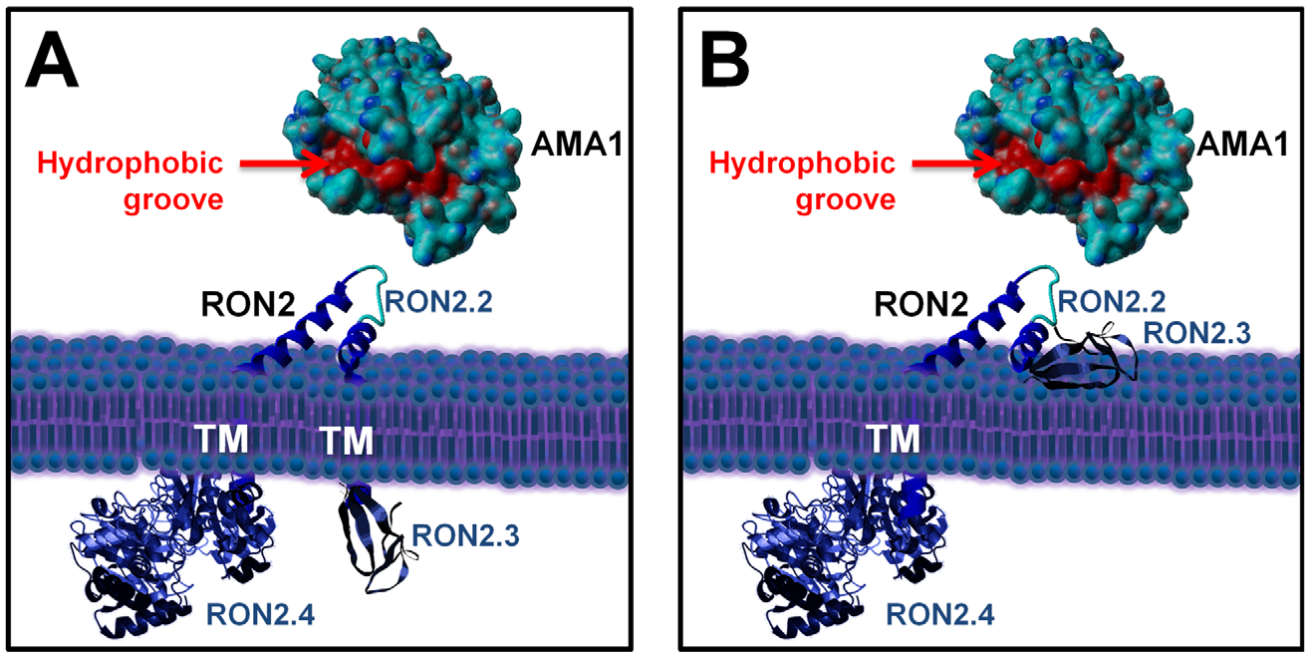
Current alternative models of the RON2-AMA1 interaction at the MJ. (A) with TgRON2 C-terminal domain exposed inside the host cell; (B) with TgRON2 C-terminal domain exposed outside. TgAMA1 ectodomain was rendered from structural data ([20], (PDB ID: 2X2Z)) using Yasara view software (www.yasara.org) with molecular surface rendering. Amino acids from the hydrophobic groove region are coloured in red. No structure currently exists for TgRON2, but the protein was grossly schematized based on secondary structure predictions.

Overall, this has led us to precise RON2 topology at the host cell plasma membrane, as we have now identified the region of RON2 interacting with AMA1 (presumably exposed outside the host cell) and another region localized inside the host cell (Figure 8). However, the functionality of the last predicted TM domain and the disposition of the well conserved C-terminal domain are still under debate, as it could be either exposed inside, or outside the host cell (Figure 8, A and B).

AMA1 is known to play a key role in invasion by Apicomplexa parasites. For instance, it appears to be essential for *Plasmodium* blood stage invasion, as no AMA1 knock-out mutants could be obtained for this stage [11] and it is also essential for invasion by *T. gondii* tachyzoites [10]. This micronemal protein, expressed at the surface of the parasite, is a promising vaccine candidate for malaria [13] and has hence been extensively studied in *Plasmodium*, especially regarding binding regions of inhibitory antibodies. Some antibodies bind regions of the *P. falciparum* AMA1 ectodomain that are functionally important: for instance, MAb 4G2 has been shown to block formation of a functional complex between PfAMA1 and other components of the MJ, without identifying precisely the AMA1-interacting protein [14]. The interaction domain of PfAMA1 with other members of the complex seems to be located within a particular hydrophobic groove located in the ectodomain of PfAMA1. Similarly, inhibitory AMA1-binding peptides that block *P. falciparum* merozoite invasion of the erythrocyte have been described, such as peptide R1 [30], which has been recently shown to bind the hydrophobic groove of PfAMA1 and to prevent the association of this protein with other MJ partners [23]. Here, we have also evaluated the intergeneric conservation of the RON2-AMA1 interaction by performing ELISA binding assays between recombinant PfAMA1 ectodomain and recombinant PfRON2-5, similar to what we had demonstrated with their *Toxoplasma* counterparts. Although both proteins display a significant degree of sequence variation between these two Apicomplexa genera, the interaction was found to be strong and specific (highlighted by the fact that the recombinant PfRON2 domain was binding strongly to PfAMA1, but not to recombinant AMA1 from another *Plasmodium* species or from *Toxoplasma*). Moreover, the role of this interaction for *P. falciparum* merozoites and for *T. gondii* tachyzoites also appears to be similar, as in both cases respective C-terminal RON2 recombinant proteins had a blocking effect on host cell invasion. Thus, the RON2-AMA1 interaction is evolutionarily conserved, reflecting a co-evolution of AMA1 protein with its RON2 interaction partner and underlining its important functional role. Moreover, we show that the R1 peptide is a competitive inhibitor for the PfAMA1-PfRON2 interaction. R1 has been shown to bind the PfAMA1 hydrophobic trough and to block invasion after the initial attachment to the erythrocyte and reorientation of the merozoite towards its apical pole. We propose that the RON2 sequence located between the two C-terminal TM domains could be structured as a loop (the sequence contains two conserved cysteines that could be forming a disulphide bridge) and interacts with AMA1 at the hydrophobic groove that has been identified as important for interaction with MJ partners in *Plasmodium*. Interestingly, the recent crystal structure of TgAMA1 has revealed that this hydrophobic trough is also conserved in *Toxoplasma* [20].

AMA1 is a leading malaria vaccine candidate, inducing antibodies that inhibit invasion and confer protection in animals. The sequence of AMA1 is rather conserved among various *Plasmodium* spp., with the level of amino acid sequence identity exceeding 50% in pairwise comparisons among all known sequences. Strain variability in *Plasmodium* AMA1, however, is a cause of concern for vaccinologists [13]. Indeed, the protective response induced by AMA1 has been shown to be strain-specific [28], largely as a consequence of the polymorphism of the regions surrounding the hydrophobic trough [19] that binds to the RON complex [14]. For example, the inhibitory effect of peptide R1 is strain-specific, binding to PfAMA1 3D7 but not to its counterpart from W2mef strain. By contrast, we have shown here that the PfRON2-5 3D7 recombinant protein binds to PfAMA1 from strains 3D7 and FVO, which suggests that the interaction is not strain-dependent, although further studies will be necessary to validate this apparent conservation. AMA1 and other MJ components play an essential role in invasion by Apicomplexa and for their virulence. Mapping the residues of AMA1 and RON2 involved in the interaction and determining the conformation they adopt upon interaction by co-crystallization and structural determination should help designing small inhibitory molecules targeting the conserved hydrophobic trough and thereby minimize the risk of evasion by the parasite. This work is a first step towards dissecting the specific interaction between two members of the MJ protein complex but, more generally, deciphering the MJ interactome will be important to define the global molecular architecture of this structure. Anchoring of the MJ on the host cell and links on the parasite side with the motor complex are of particular interest to understand how such a structure articulates and moves along the parasite during invasion.

## Materials and Methods

### Ethics statement

This study was conducted according to European Union guidelines for the handling of laboratory animals and the immunization protocol for antibody production in rabbits was conducted at the CRBM animal house (Montpellier) and approved by the Committee on the Ethics of Animal Experiments (Languedoc-Roussillon, Montpellier) (Permit Number: D34-172-4, delivered on 20/09/2009).

### Host cells and parasite cultures

All *T. gondii* tachyzoites were grown in human foreskin fibroblasts (HFF) or Vero cells grown in standard conditions. Tachyzoites of the RH *hxgprt-* strain of *T. gondii* deleted for hypoxanthine guanine phosphoribosyl transferase (ΔHX strain) [31] or KOi AMA1 [10] were used throughout the study. *P. falciparum* 3D7 strain was cultured in human red blood cells (EFS, Pyrénées Méditerranée, France) at 5% hematocrit in medium composed of RMPI 1640 containing 25 mM HEPES, 10% human serum, and incubated at 37°C [32] in a tri-gas mix of 5% O_2_, 5% CO_2_ and 90% N_2_.

### Molecular cloning and production of recombinant proteins

The primers used for constructions are listed in Table S2.

Plasmid pcDNA3-TgAMA1 was designed to express the TgAMA1 protein in BHK-21 cells. It was constructed using plasmid pMAH14-AMA1, in which the two putative N-glycosylation sites (Asn at position 86 and 421 in AMA1 sequence) were replaced respectively by two alanine residues using two steps of site directed mutagenesis. We used a natural NheI site present at position 193, after the cleavage site of the pro-peptide of TgAMA1 to clone the cDNA corresponding to the mature form of TgAMA1 (GenBank AF010264) in pcDNA3.1 The NheI/EcorI fragment of pMAH14-AMA1 was cloned in pcDNA3.1 (Invitrogen) and next the signal peptide from TgMIC3, PCR-amplified from pSS-MIC3 with primers ML5/ML6 [24], was inserted into the NheI site. Compatible NheI and XbaI sites are present in ML5 and ML6, respectively.

Plasmid pcDNA3-PfAMA1 was designed to express the PfAMA1 protein in BHK-21 cells. Generation of a complete synthetic variant gene of Pf3D7 AMA1 utilizing mouse codon usage and lacking putative N-glycosylation sites as previously described [17] was performed by Genscript, USA. Synthetic Pf3D7-AMA1 gene was recovered from the pUC57 vector using XbaI and HindIII restriction sites and subsequently cloned in the mammalian expression vector pcDNA3.1/myc-His(A) (Invitrogen), thereby enabling an in frame C-terminal fusion of PfAMA1 with myc epitope.

Plasmid pGEX-TgRON2-2 was designed to express a N-terminal GST-tagged recombinant protein TgRON2 corresponding to the region between TM2 and TM3 and extending into 14 residues of TM3 (V_1295_ to P_1359_) as described previously [5].

Plasmid pGEX-TgRON2-5 was designed to express a N-terminal GST-tagged recombinant protein TgRON2 corresponding to the region between TM2 and TM3 (V_1295_ to P_1347_). Corresponding TgRON2 fragment was obtained by PCR from TgRON2 cDNA with primers ML224 and ML473.

Plasmid pET24a-TgRON2-3 was designed to express a TgRON2 domain corresponding to the last 111 residues of the protein and pET24a-TgRON2-4 was designed to express a TgRON2 domain corresponding to residues 25 (starting after predicted signal peptide sequence) to 288. The corresponding DNA sequences were PCR-amplified from *T. gondii* TgRON2 cDNA (GenBank HQ110093) using primers ML333/ML334 and ML398/ML399, respectively, and cloned between NdeI and XhoI in pET24a (Merck Biosciences) allowing expression of C-terminal His-tagged recombinant proteins.

Plasmid pET22b-TgAMA1 was designed to express the full ectodomain (domains I, II, and III) for antibody production. Primers ML390 and ML391 were used on *T. gondii* tachyzoite cDNA to produce by PCR a fragment that was subsequently cloned into pET22b with NdeI and XhoI restriction enzymes. Soluble recombinant TgAMA1 for binding assays was produced as described previously [20].

Plasmid pGEX-PfRON2-5 was designed to express a Pf3D7-RON2 domain corresponding to the region between TM2 and TM3 (M_2020_ to K_2067_). The corresponding DNA was PCR amplified from *P. falciparum* genomic DNA using primers ML465 and ML495 and cloned between EcoRI and BamHI in pGex-4T-3 (GE Healthcare Life Sciences), allowing expression of N-terminal GST-tagged recombinant protein.

All the constructs, except pGEX-PfRON2-5, were transformed into *E. coli* C41 cells to produce recombinant protein. Expression of PfRON2-5 was done in BL21pRIG *E. coli* cells that contain pRIG plasmid encoding 3 tRNAs (Arg, Ile and Gly) that recognize codons abundant in *P. falciparum* genes and rarely used by *E. coli*.

His-tagged and GST-tagged proteins were purified by affinity purification on nickel- or gluthatione-agarose column in native (TgRON2-2, TgRON2-3, TgRON2-5 and PfRON2-5) or denaturing condition (TgAMA1, TgRON2-4) and according to the manufacturer's instructions. Recombinant proteins were used to raise polyclonal antibodies in rabbit as described previously [5]. Before rabbit immunization, a further purification of TgRON2-4 was achieved by electroelution under denaturing conditions. For binding assays, recombinant proteins were dialyzed against PBS pH7, concentrated on Amicon Ultra 3K and quantified by Pierce BCA protein assay.

Recombinant *Plasmodium* AMA1 ectodomains were produced in *Pichia pastoris* as described previously for *P. vivax* [29], and *P. falciparum* FVO and 3D7 [28]. Potential N-glycosylation sites were removed by mutagenesis.

### Cells transfection, glass beads loading of antibodies into host cells and immunofluorescence

BHK-21 cells were transfected as previously described [24]. IFA of invading parasites were obtained by synchronisation of invasion at 4°C [3] or using a K^+^ buffer shift [33]. For IFA of transfected BHK-21 cells, intracellular parasites or invading parasites, cells were fixed with 4% paraformaldehyde (PAF) in PBS for 30 min, washed and permeabilized, or not, with 0.1% Triton X100 or with 0.05% saponin in PBS for 10 min, blocked with 10% fetal bovine serum in PBS (PFBS) for 30 min, incubated with primary antibodies diluted in 2% PFBS, washed and then incubated with secondary antibody coupled to Alexa 594 or Alexa 488 (Sigma). Finally the coverslips were washed and mounted onto microscope slides using Immumount (Calbiochem). Images were collected either i) with a Leica DMRA2 microscope equipped for epifluorescence, the images being recorded with a COOLSNAP CCD camera (Photometrics) driven by the Metaview software (Universal Imaging Co.) or ii) with a Zeiss Axioimager epifluorescence microscope and images were recorded with a Zeiss Axiocam MRm CCD camera driven by the Axiovision software (Zeiss), at the Montpellier RIO imaging facility. The antibodies used and their dilution were the following: mouse monoclonal antibodies (MAb) anti-TgAMA1 CL22 (1∶1000, anti-cytosolic domain) [7] and B3.90 (1∶300, anti-ectodomain) [6], mouse MAb anti-RON4 T54H1 undiluted hybridoma culture supernatant [34], rabbit anti-TgAMA1 (1∶1000, anti-ectodomain, this work), rat anti-RON2-2 (anti-RON2c, 1∶1000) [5], rabbit anti-RON2-1 (anti-RON2n, 1∶1000) [5], rat anti-RON8 (1∶500) [5], rabbit anti-RON2-3 (1∶200) (this work), rabbit anti-RON2-4 (1∶1000) (this work), rabbit anti-MIC3 (1∶1000) [35], rabbit anti-MIC8 (1∶500) [36], mouse MAb anti-*P. vivax* AMA1 F8.12.19 at 1 µg/ml [26], commercial mouse MAb anti-cMyc 9E10 (1∶400) or rabbit anti-cMyc (A-14) antibodies (Santa Cruz Biotechnology) and rat anti-GST antibodies (1∶300).

Rabbit anti-RON2-1 (anti-RON2n) [5], rat anti-RON2-2 (anti-RON2c) [5] and rabbit anti-RON2-3 (this work) were further affinity-purified on an antigen strip excised from a nitrocellulose protein blot. Briefly, ∼500 µg of respective purified recombinant proteins were separated by SDS-PAGE and transferred onto nitocellulose blot. Proteins were labeled with Ponceau red staining and the corresponding strip was excised. Strips were saturated in PBS+1% BSA (w/v) for one hour. 250 µl of immune sera were adsorbed on their respective recombinant proteins for 2 hours. Following extensive washes in PBS, specific antibodies were eluted in 1 ml of 100 mM glycine pH 2.5 buffer and immediately neutralized with one-tenth volume of 1 M Tris-HCl pH 8.8.

Loading of antibodies into host cells by the glass bead permeabilization method was performed as described previously [5].

### RON2-AMA1 interaction tests

#### Co-immunoprecipitation

Parasite solubilization in 1% NP40 or in 0.6% SDS and immunosorption procedures were done as described previously using rat anti-RON2-2 or rabbit anti-RON2-1 [3]. Elution from beads was performed during 5 min at 95°C with SDS-PAGE sample buffer and co-immunoprecipitation of AMA1 was detected using MAb CL22.

#### Expression in BHK-21 cells

Overnight transfected cells were washed in HBSS (Invitrogen) before addition of TgRON2-2 or PfRON2-5 diluted in HBSS at concentrations ranging from 0.1 µg/ml to 10 µg/ml, or 20 µg/ml, respectively, for 1 h. After five washes in PBS to remove unbound protein, cells were fixed in 4% PAF and further processed for IFA as described above.

#### ELISA

100 µl of recombinant AMA1 protein diluted in 1 M sodium-carbonate buffer pH 9.6 was coated at 1 µg/ml in Maxisorp 96 wells plates (Nunc) and incubated overnight at 4°C with gentle agitation. Unbound protein was removed by washing in PBS, 0.05% Tween 20. Saturation was performed in blocking buffer (PBS, 1% bovine serum albumin, 0.02% sodium azide) for 1 h. 100 µl of recombinant RON2 protein diluted in blocking buffer was added to each well, and incubated 1 h with shaking. Bound RON2 protein was detected using rat anti-GST (1∶1000) followed by an anti-rat IgG horseradish peroxidase conjugate (1∶2500) (ZyMAX, Invitrogen). Binding was visualized using SIGMAFAST-OPD (o-phenylenediamine) tablet (Sigma Aldrich) and absorbance read at 450 nm.

Competitive ELISA assays were essentially carried out as described above, except that increasing concentrations of either competitive *P.falciparum* R1 peptide [30] (VFAEFLPLFSKFGSRMHILK, PolyPeptide Laboratories, France) or *T.gondii* control peptide P8 (SAPQAIAKATTS, Peptide 2.0, USA) corresponding to a variable part of RON2-2, were pre-incubated for 15 min with recombinant protein before being added to each well.

#### Far western

*T. gondii* tachyzoites were lysed for 30 min at 37°C in a non-reducing SDS sample buffer and separated on 10% polyacrylamide gel (∼5.10^6^ cells per lane). Proteins were electrotransferred on nitrocellulose filters (Schleicher and Schuell). Membranes were washed twice in PBS and incubated for 2 hours at room temperature in PBS containing 1% bovine serum albumin and 5% non-fat milk. Membranes were incubated with GST or TgRON2-2 at 100 µg/ml in PBS for 2 h, washed 6 times in PBS for 10 min each and then probed as described previously [3] with rat anti-GST (1∶300), followed by goat anti-rat IgG alkaline phosphatase conjugate (Promega). Mouse monoclonal anti-SAG1 (T4 1E5) diluted 1∶1000 was used as a loading control [37].

#### In situ recombinant protein binding

Confluent HFF monolayers grown on coverslips were infected with RH tachyzoites for 18 h and fixed for 30 min in 4% PAF in PBS. After permeabilization with 0.1% Triton X100 and saturation with 10% PFBS for 30 min, the cells were incubated for one hour with with 1 µg/ml of GST or TgRON2-2, washed and proceed as described previously for IFA using anti-GST.

### Invasion assays

#### *T. gondii*

Freshly released tachyzoites (1.10^6^) were pre-incubated 30 minutes at 37°C with 200 µg/ml (6 µM) of recombinant RON2 proteins prior to addition onto confluent HFFs grown in 16 wells Lab-Tek chamber slides (Nunc). Invasion was allowed to take place during 30 minutes before extensive washes in PBS buffer. 5 minutes synchronized invasions were performed as described above at 4°C. IFA analysis was subsequently performed as described previously.

#### *P. falciparum*

*P. falciparum* 3D7 parasites were sorbitol-synchronyzed [38] prior to schizonts stage enrichment *via* the magnetic method using MACS system (Miltenyi Biotec) [39]. 100 µl of infected red blood cells (2% parasitemia, 0.75% haematocrit) were mixed with 100 µl of R1 peptide or recombinant protein diluted in RPMI 1640 supplemented with human serum. R1 peptide concentrations were 10 µg/ml (4.3 µM) and 50 µg/ml (21.7 µM) and equivalent molar concentrations of PfRON2-5 and GST were used. Control blood smears were performed at regular time points to check for proper merozoites re-invasion. After 4–6 hours, most of the schizonts had ruptured and free merozoites had already re-invaded. To facilitate counting, blood smears were also collected 16 hours post-invasion and used for ring-stage parasites counting.

## Supporting Information

Figure S1 Co-localisation of TgRON2 with TgRON4 at the residual junction (arrowhead) after completion of invasion. Monoclonal T54H1 anti-RON4 antibody (green) and rabbit polyclonal anti-RON2-3 and RON2-4 (red) antibodies were used.
(2.50 MB TIF)

Figure S2 Alignment of RON2 orthologues from different Apicomplexan species (see Table S1 for database accession numbers). Amino acid sequences were aligned using the MUSCLE algorithm with Geneious software (www.geneious.com). Predicted TM1, TM2 and TM3 (see Table S1) are annotated in red. Yellow arrows on top of *T. gondii* and *P. falciparum* RON2 sequences correspond to TgRON2-2 (long), TgRON2-5 (short) and PfRON2-5 recombinant proteins, which are binding to AMA1. Global sequence conservation is depicted under consensus sequence with bars and colour scale (green: highly conserved, red: less conserved). Background color for amino acids indicates similarity, with a grayscale ranging from 100% similar (black) to less than 60% similar (white).
(2.10 MB PDF)

Figure S3 Controls for specific binding of TgRON2-2 to Tg AMA1. (A) GST does not bind to AMA1-expressing BHK-21 cells. BHK-21 cells were incubated with 10 µg/ml of GST. TgAMA1 was revealed with anti-ectodomain B3.90 antibody. TgRON2-2 binding is specific to the expression of TgAMA1 at the surface of BHK-21 cells. (B) BHK-21 cells were transfected to express unrelated TgMIC8 protein at their surface (revealed with specific antibody) and they were incubated with of 10 µg/ml TgRON2-2. In both cases cells were not permeabilized, to show surface labeling, and anti-GST antibody was used for detection of the recombinant proteins.
(4.13 MB TIF)

Figure S4 Use of glass bead cell antibody loading method to assess the intracellular exposure of TgRON2 domains. HFF cells were pre-loaded with antibodies directed against TgRON2-2 or TgRON2-3 and were pulse-infected for 2.5 min, followed by IFA. The upper series shows progressing invasions while the lower series shows terminating invasions, in both cases the arrowhead indicates the MJ and magnifications are shown on the right. Scale bar = 5 µm.
(9.48 MB TIF)

Figure S5 Incubation with TgRON2-2 does not prevent attachment of *T. gondii* tachyzoites. Attachment of RHΔ*hxgprt* (dark grey) or KOi AMA1 (light grey) strains to HFF cells fixed with 0.075% glutaraldehyde, in presence of GST (plain bars) or TgRON2-2 (dashed bars). Data presented here are the means ±SD, representative of five independent experiments done in triplicate.
(1.16 MB TIF)

Figure S6 *Plasmodium* PfRON2-5 does not inhibit the invasion of HFF cells by *T. gondii*. (A) Schematic representation of the corresponding RON2 recombinant proteins used. (B) TgRON2-5 can bind efficiently to TgAMA1. ELISAs were carried out with increasing concentrations of Pf3D7-RON2-5 (purple), TgRON2-2 (green), TgRON2-5 (red) or GST (black) on recombinant TgAMA1 protein coated at 1 µg/ml. (C) Extracellular KOi AMA1 tachyzoites were pre-incubated with either GST, PfRON2-5, TgRON2-2 or TgRON2-5 at 200 µg/ml (6 µM), before being added to confluent HFF monolayers for 30 min. Significant decrease in *T.gondii* invasion (t-test, ** p<0.01) was specifically observed in the presence of TgRON2-2 and TgRON2-5.
(0.74 MB TIF)

Table S1 Prediction of the conservation of three transmembrane domains (TM1, TM2, TM3, see Figure S2) in apicomplexan RON2 orthologues using different software: TMHMM (http://www.cbs.dtu.dk/services/TMHMM/), TOP PRED (http://mobyle.pasteur.fr/cgi-bin/portal.py?form=toppred), SOSUI (http://bp.nuap.nagoya-u.ac.jp/sosui/sosui_submit.html), HMMTOP (http://www.enzim.hu/hmmtop/) and CONPRED II (http://bioinfo.si.hirosaki-u.ac.jp/~ConPred2/).
(0.03 MB XLS)

Table S2 Primers used in this study. Restriction sites used for cloning are underlined.
(0.03 MB XLS)
