# Supplementary figures and images for: The RON2-AMA1 Interaction is a Critical Step in Moving Junction-Dependent Invasion by Apicomplexan Parasites
Source: PLoS Pathog. 2011 Feb 10;7(2):e1001276. doi: 10.1371/journal.ppat.1001276 (PMC3037350; doi:10.1371/journal.ppat.1001276)

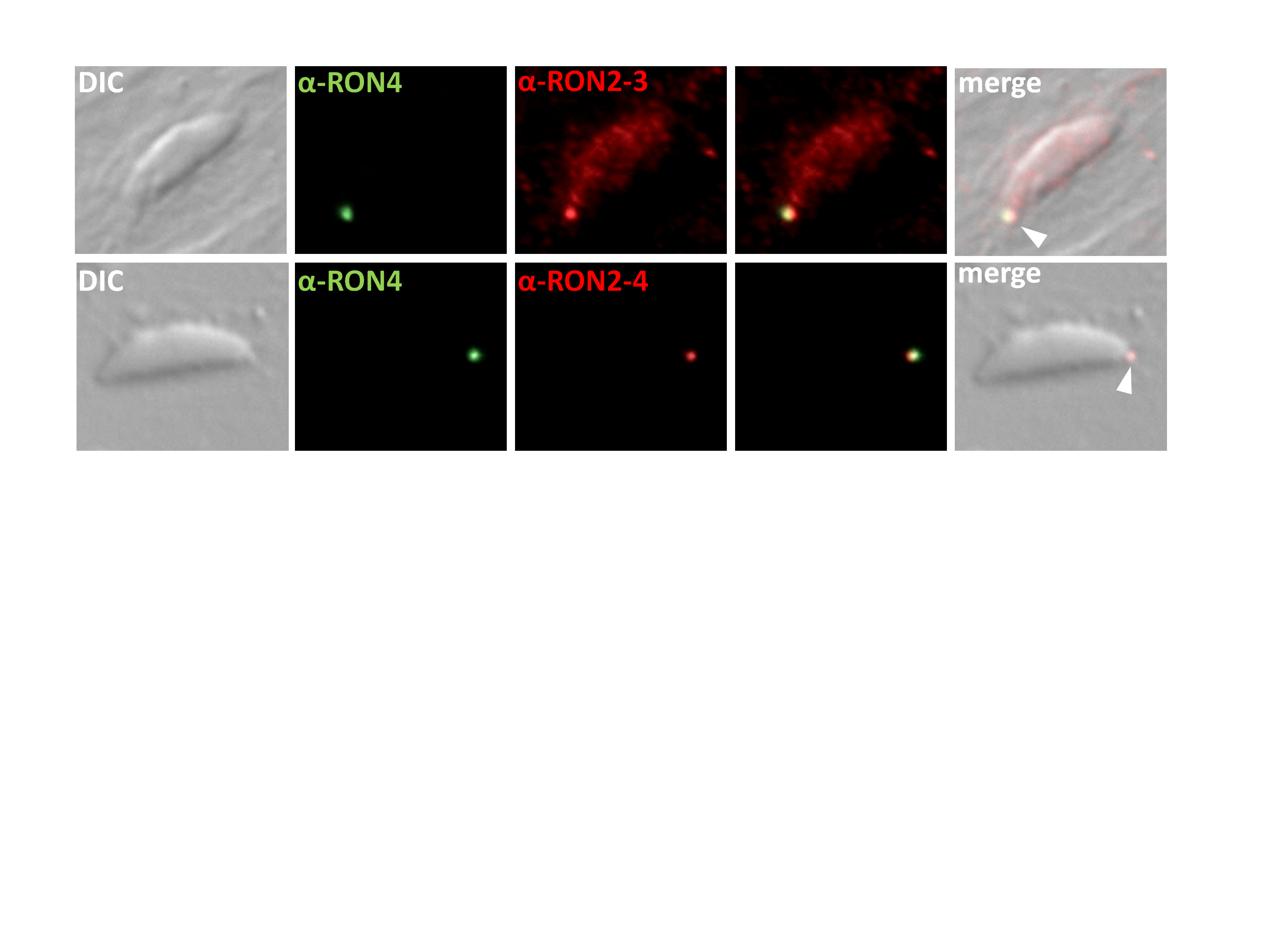

Supplement: Figure S1 — Co-localisation of TgRON2 with TgRON4 at the residual junction (arrowhead) after completion of invasion. Monoclonal T54H1 anti-RON4 antibody (green) and rabbit polyclonal anti-RON2-3 and RON2-4 (red) antibodies were used. (2.50 MB TIF) [file ppat.1001276.s001.tif]

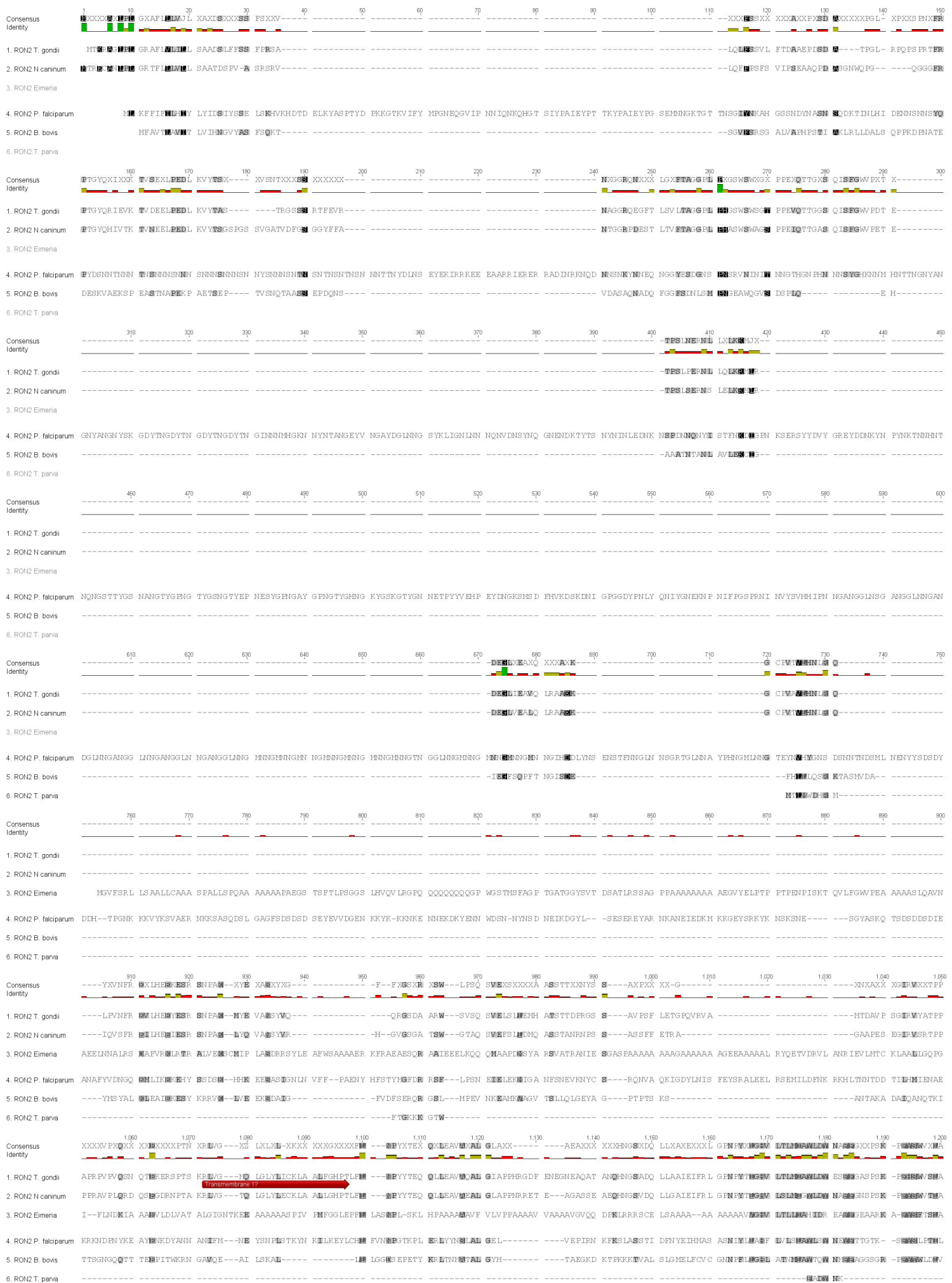

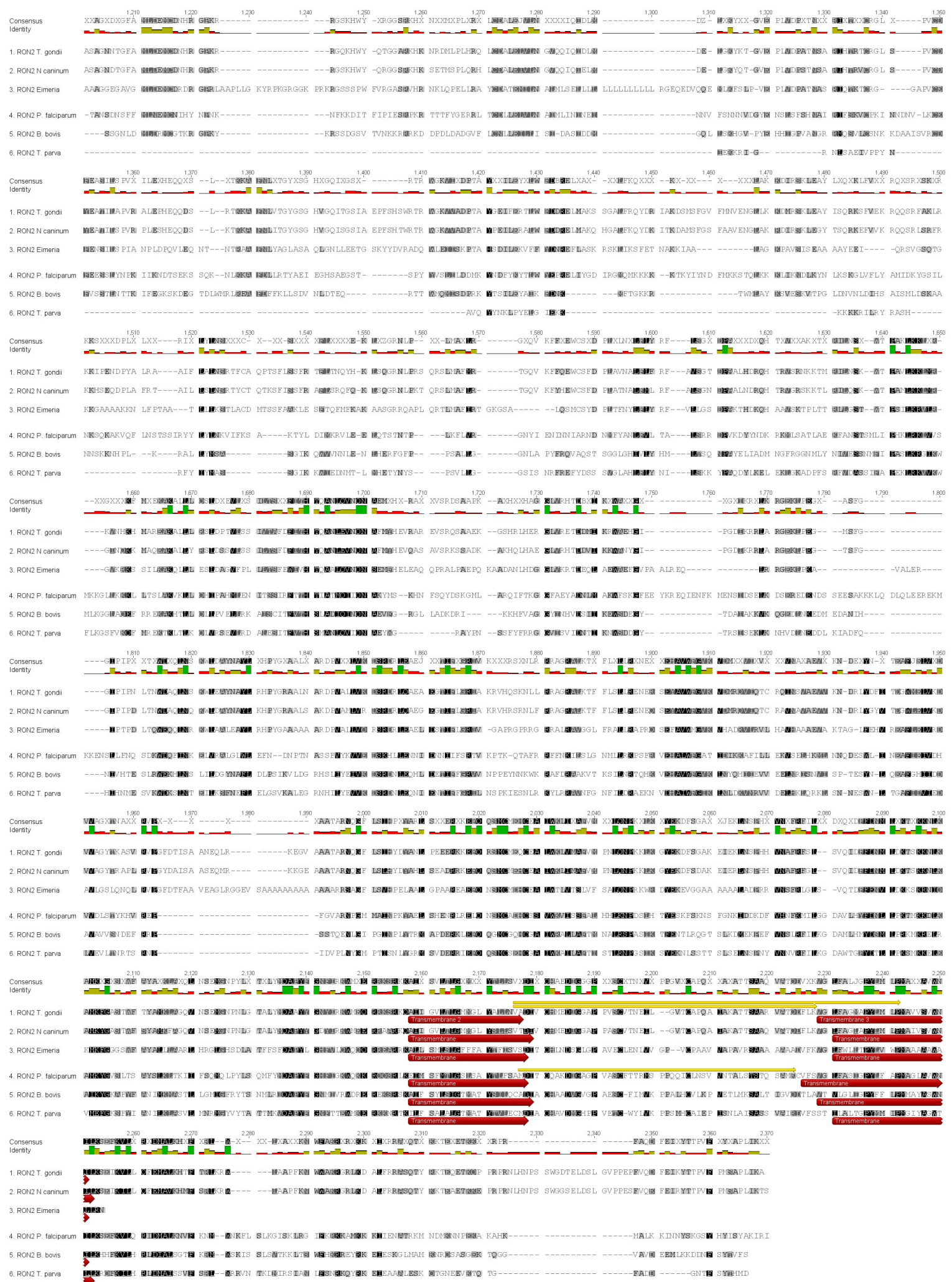

Supplement: Figure S2 — Alignment of RON2 orthologues from different Apicomplexan species (see Table S1 for database accession numbers). Amino acid sequences were aligned using the MUSCLE algorithm with Geneious software (www.geneious.com). Predicted TM1, TM2 and TM3 (see Table S1) are annotated in red. Yellow arrows on top of T. gondii and P. falciparum RON2 sequences correspond to TgRON2-2 (long), TgRON2-5 (short) and PfRON2-5 recombinant proteins, which are binding to AMA1. Global sequence conservation is depicted under consensus sequence with bars and colour scale (green: highly conserved, red: less conserved). Background color for amino acids indicates similarity, with a grayscale ranging from 100% similar (black) to less than 60% similar (white). (2.10 MB PDF) [file ppat.1001276.s002.pdf]

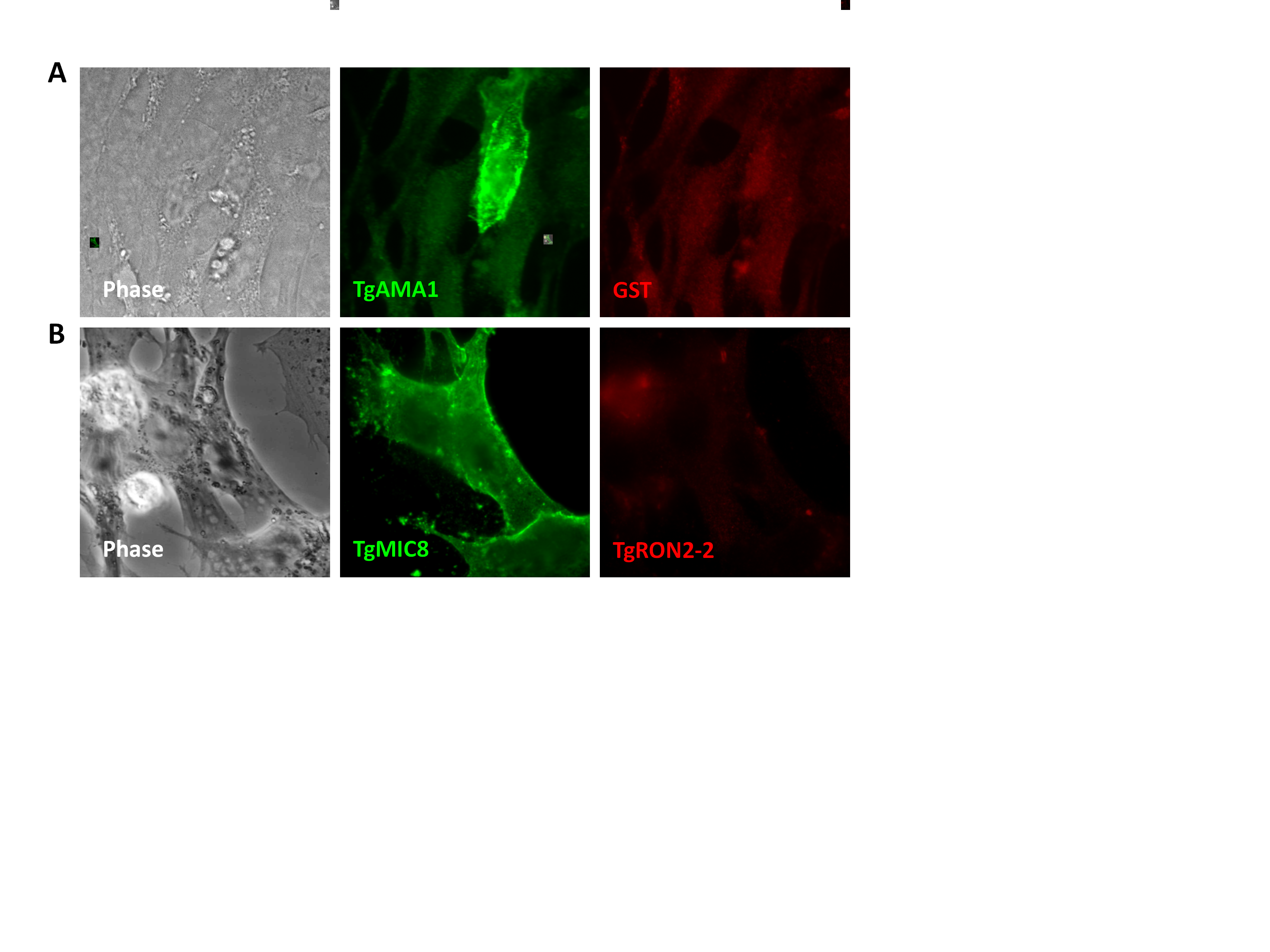

Supplement: Figure S3 — Controls for specific binding of TgRON2-2 to Tg AMA1. (A) GST does not bind to AMA1-expressing BHK-21 cells. BHK-21 cells were incubated with 10 µg/ml of GST. TgAMA1 was revealed with anti-ectodomain B3.90 antibody. TgRON2-2 binding is specific to the expression of TgAMA1 at the surface of BHK-21 cells. (B) BHK-21 cells were transfected to express unrelated TgMIC8 protein at their surface (revealed with specific antibody) and they were incubated with of 10 µg/ml TgRON2-2. In both cases cells were not permeabilized, to show surface labeling, and anti-GST antibody was used for detection of the recombinant proteins. (4.13 MB TIF) [file ppat.1001276.s003.tif]

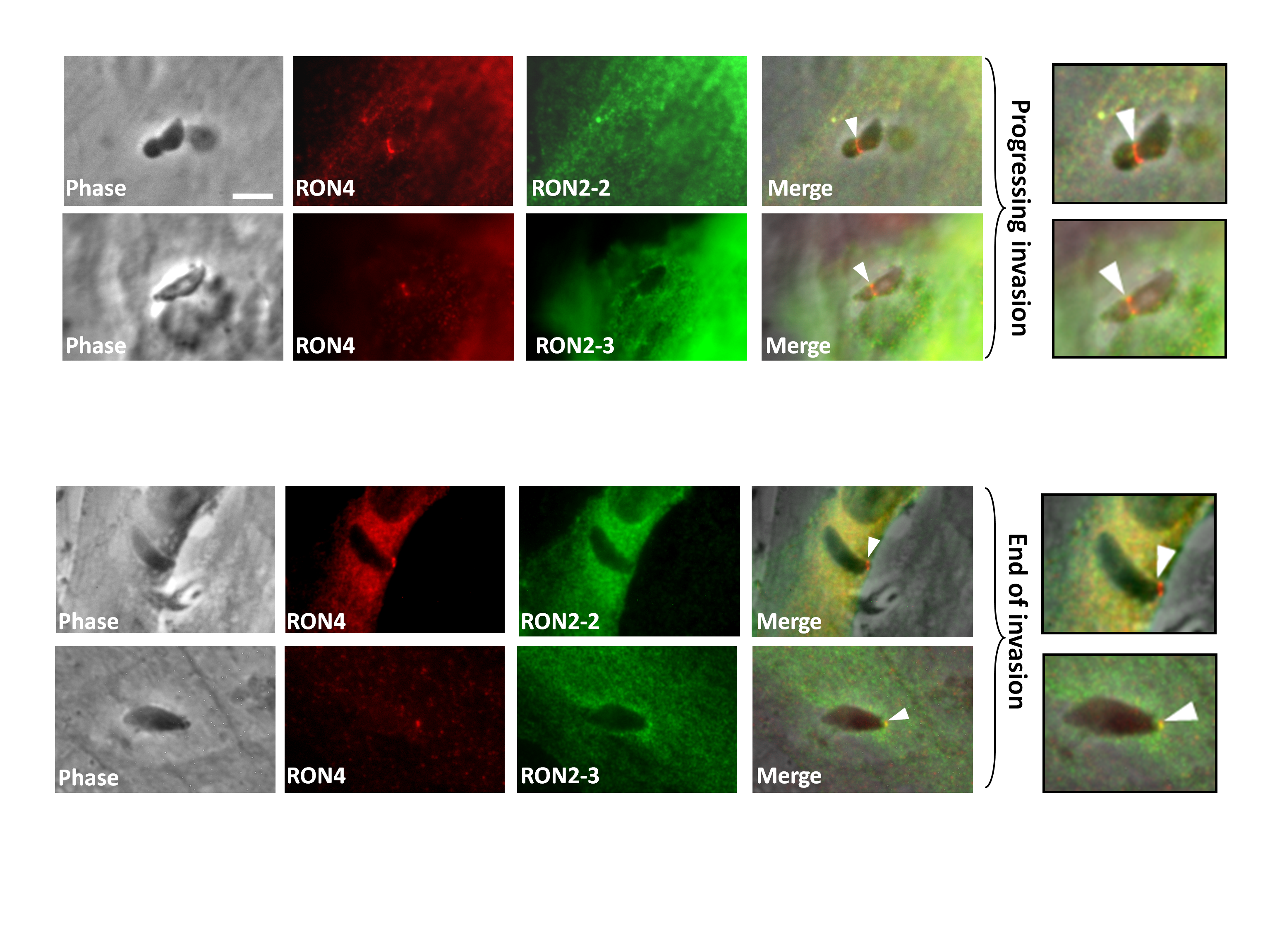

Supplement: Figure S4 — Use of glass bead cell antibody loading method to assess the intracellular exposure of TgRON2 domains. HFF cells were pre-loaded with antibodies directed against TgRON2-2 or TgRON2-3 and were pulse-infected for 2.5 min, followed by IFA. The upper series shows progressing invasions while the lower series shows terminating invasions, in both cases the arrowhead indicates the MJ and magnifications are shown on the right. Scale bar = 5 µm. (9.48 MB TIF) [file ppat.1001276.s004.tif]

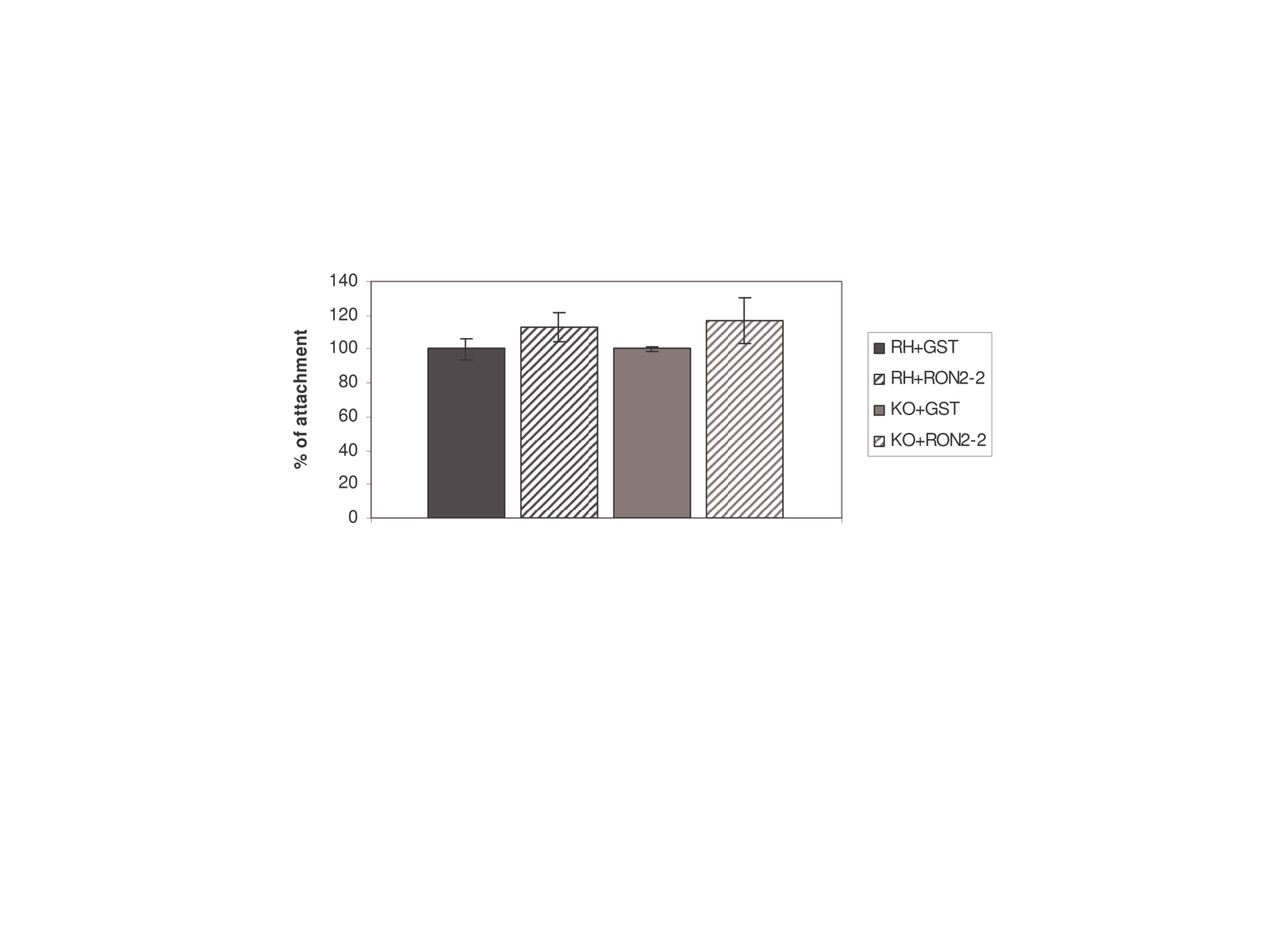

Supplement: Figure S5 — Incubation with TgRON2-2 does not prevent attachment of T. gondii tachyzoites. Attachment of RHΔhxgprt (dark grey) or KOi AMA1 (light grey) strains to HFF cells fixed with 0.075% glutaraldehyde, in presence of GST (plain bars) or TgRON2-2 (dashed bars). Data presented here are the means ±SD, representative of five independent experiments done in triplicate. (1.16 MB TIF) [file ppat.1001276.s005.tif]

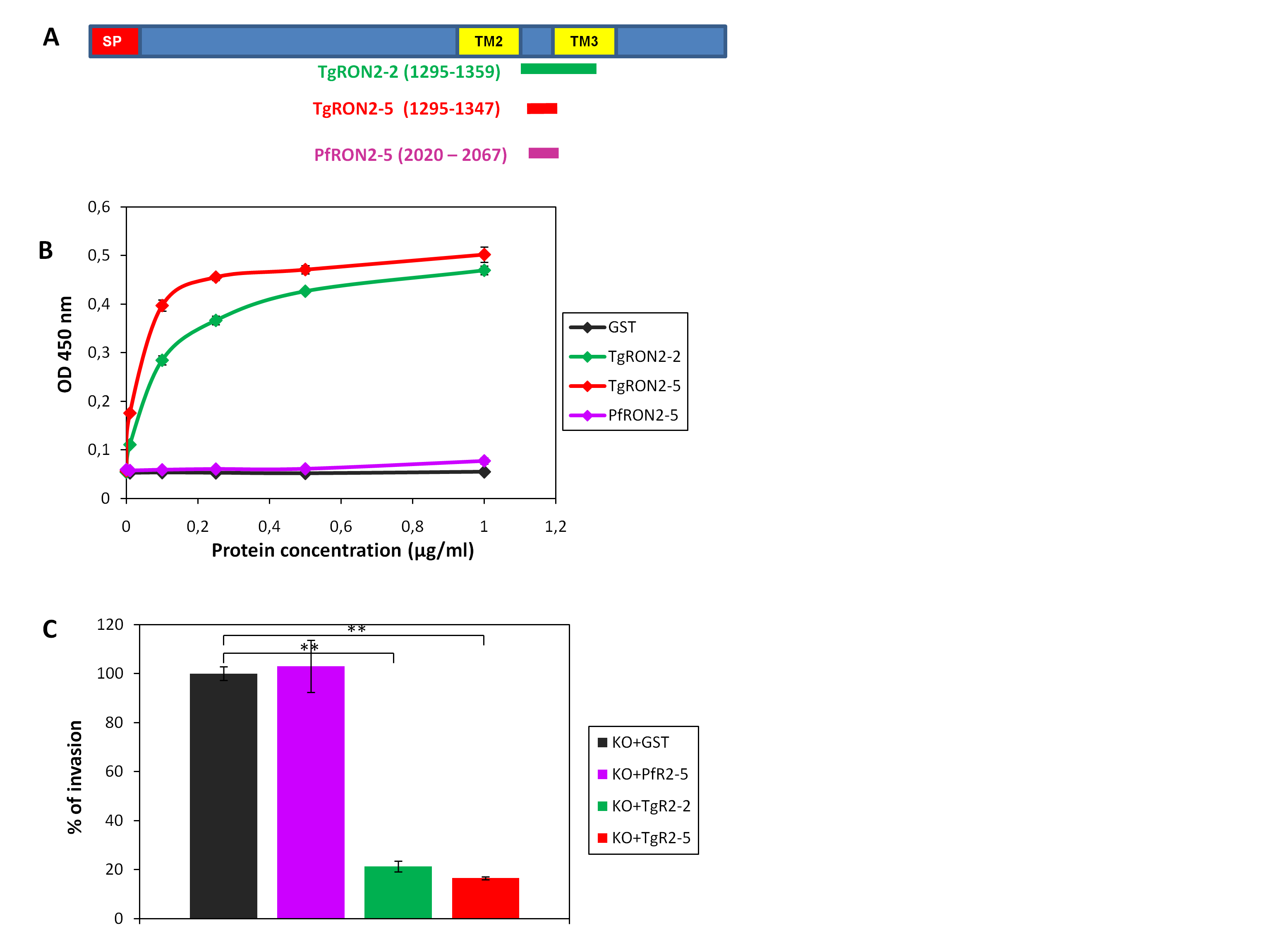

Supplement: Figure S6 — Plasmodium PfRON2-5 does not inhibit the invasion of HFF cells by T. gondii. (A) Schematic representation of the corresponding RON2 recombinant proteins used. (B) TgRON2-5 can bind efficiently to TgAMA1. ELISAs were carried out with increasing concentrations of Pf3D7-RON2-5 (purple), TgRON2-2 (green), TgRON2-5 (red) or GST (black) on recombinant TgAMA1 protein coated at 1 µg/ml. (C) Extracellular KOi AMA1 tachyzoites were pre-incubated with either GST, PfRON2-5, TgRON2-2 or TgRON2-5 at 200 µg/ml (6 µM), before being added to confluent HFF monolayers for 30 min. Significant decrease in T.gondii invasion (t-test, ** p<0.01) was specifically observed in the presence of TgRON2-2 and TgRON2-5. (0.74 MB TIF) [file ppat.1001276.s006.tif]
